# *Polygonatum cyrtonema* Polysaccharide Ameliorates Functional Constipation in Mice via the Gut Microbiota–Pentadecanoic Acid–GPR40 Axis

**DOI:** 10.1021/acs.jafc.6c06937

**Published:** 2026-10-20

**Authors:** Xiaoying Liu, Zhongyu Zhang, Yuanyuan Fang, Beixin Yuan, Huilong Lian, Wenxin Yin, Jinming Zhang, Qin Yuan, Shengpeng Wang

**Affiliations:** † State Key Laboratory of Mechanism and Quality of Chinese Medicine, 59193 University of Macau, Macao SAR 999078, China; ‡ School of Pharmacy, 118385 Chengdu University of Traditional Chinese Medicine, Chengdu 611137, China; § Macao Centre for Research and Development in Chinese Medicine, Institute of Chinese Medical Sciences, University of Macau, Macao SAR 999078, China

**Keywords:** Constipation, Gut microbiota, Polygonatum cyrtonema Polysaccharide, Pentadecanoic acid, GPR40

## Abstract

Constipation is a widespread gastrointestinal dysfunction that severely diminishes life satisfaction, yet current pharmacological interventions frequently carry risks of dependency. This study investigates the therapeutic potential and underlying mechanism of*Polygonatum cyrtonema* polysaccharide (PCP), an inulin neoseries-type fructan, in mitigating loperamide-induced constipation in mice. Our results demonstrated that PCP treatment significantly normalized defecation indicators, including increased fecal water content and accelerated small-intestinal transit. Fecal microbiota transplantation (FMT) confirmed that the anti-constipation efficacy of PCP was driven by the gut microbiota. Integrated 16S rRNA sequencing and metabolomic profiling revealed that PCP remodeled the gut microbiota, particularly enriching *Lachnospiraceae*, which subsequently drove the accumulation of pentadecanoic acid (PDA). Mechanistically, PDA served as a key microbial metabolite that enhanced colonic motility, likely by activating the GPR40 receptor. These findings identify the “PCP–microbiota–PDA–GPR40” axis as an novel pathway for alleviating functional constipation, supporting PCP as a promising prebiotic candidate or functional food ingredient.

## Graphical abstract

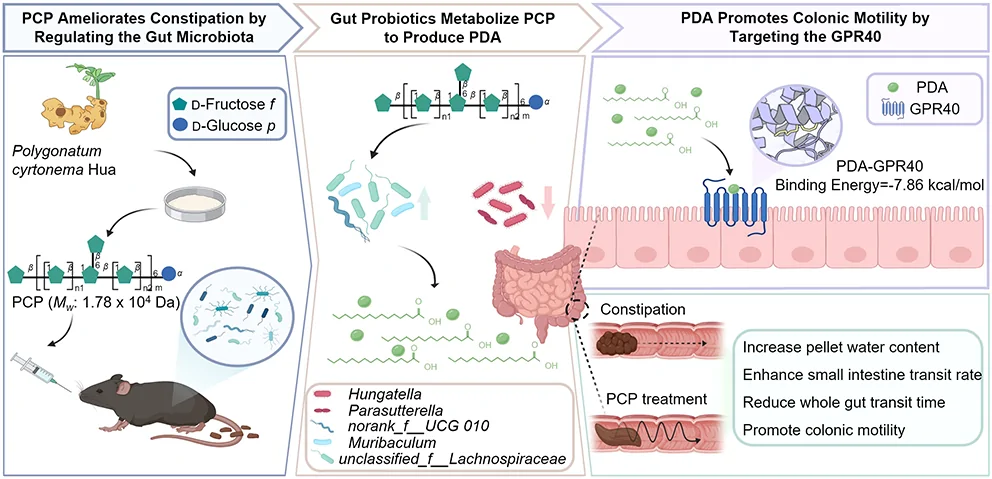

## 1 Introduction

Constipation is a considerable public health issue globally, unfavorably influencing the life satisfaction of patients and adding to the financial strain.^1^ It occurs in nearly 11–20% of adults each year and is characterized by hard or lumpy stools, along with infrequent or difficult defecation.^2,3^ Constipation can be categorized as either primary, encompassing normal transit, slow transit, and evacuatory disorders, or secondary, resulting from medication use or, uncommonly, anatomical abnormalities.^4^ Clinically, initial management involves dietary and lifestyle modifications, followed by the use of laxatives and enemas if necessary.^5^ However, these approaches often yield limited efficacy or are associated with high recurrence rates.^6^ Long-term reliance on laxatives can lead to dependency and impaired bowel function. Moreover, laxatives may cause various adverse effects, such as diarrhea, gastric discomfort, abdominal spasms, emesis, arrhythmias, myocardial infarction, and metabolic disturbances.^7^ Emerging evidence highlights that the gut microbiota is implicated in the pathogenesis of chronic constipation, altering intestinal metabolic profiles and impairing colonic motility.^8^ Therefore, developing novel, physiology-based agents that target the gut microbial ecosystem has become a crucial strategy to address the unmet needs of a larger patient population.

Dietary polysaccharides serve as fermentable substrates for gut microbiota and have arisen as attractive candidates for alleviating constipation through microbiota modulation.^9,10^ For instance, tea flower polysaccharides relieve constipation in the host through regulating the gut microbiota as well as reducing liver and kidney damage when used in combination with anticonstipation medications.^2^ *Spirulina platensis* polysaccharide alleviates constipation manifestations in mice by enhancing intestinal peristalsis, alleviating inflammatory responses in the intestinal villi, and modulating the gut microbiota composition, chiefly by upregulating the levels of probiotics.^11^ These results suggest that plant polysaccharides from diverse natural sources hold great potential for preventing and alleviating constipation. Among dietary polysaccharides, inulin-type fructans are selectively fermented by the gut microbiota and have demonstrated prebiotic effects in human clinical trials.^12^ In a rat constipation model, inulin increased fecal output and water content and normalized gut motility-related hormones, alongside elevated colonic short-chain fatty acid (SCFA) levels and Lactobacillus abundance.^13^ Separately, microbiota-derived fatty acids have been shown to regulate intestinal motility via G-protein-coupled receptors such as G-protein-coupled receptor 40 (GPR40), which is activated by medium- to long-chain fatty acids and promotes colonic motility through downstream Ca^2+^ signaling.^14,15^ Despite this, the specific microbiota-derived metabolites and host receptor pathways mediating the prokinetic effects of inulin-type fructans remain incompletely defined.

*Polygonatum cyrtonema* Hua, a traditional medicinal plant with dual medicinal and esculent qualities, has been utilized in China for more than two thousand years.^16^ The herb is primarily rich in polysaccharides, saponins, flavonoids, and other bioactive components,^17^ with polysaccharides being the predominant active constituents and requiring a minimum content of 7% according to the Chinese Pharmacopoeia.^18^ In our investigation, we extracted and refined a homogeneous polysaccharide from *Polygonatum cyrtonema* (PCP), which was characterized as an inulin neoseries-type fructan characterized by a molecular weight of 1.78 × 10^4^ Da.^19^ PCP has proven effective in alleviating colitis and reshaping the gut microbiota,^19,20^ implying its promise as a prebiotic. However, whether PCP can ameliorate constipation, and if so, which specific microbiota-derived metabolites and host signaling pathways are involved, remains unexplored.

On the basis of previous findings, our research investigated the mitigating efficacy of PCP on constipation in a mouse model and elucidated the function of gut microbiota in this process using fecal microbiota transplantation (FMT). Furthermore, multiomics analysis was employed to clarify the pathways connecting gut microbiota to host physiological function. We discovered that PCP promoted the enrichment of potential beneficial bacteria and suppressed the potential pathogenic bacteria. Notably, the beneficial gut microbiota, which primarily consist of *unclassified_f__Lachnospiraceae*, produced the beneficial metabolite pentadecanoic acid (PDA). Further analysis revealed that this microbiota-derived PDA targets GPR40 to accelerate colonic motility and alleviate constipation in the mice. These findings provide a novel strategy for relieving constipation and offer a theoretical basis for developing PCP as a prebiotic agent targeting gut microbiota.

## 2 Materials and Methods

### 2.1 Materials and Chemicals

Loperamide hydrochloride (CAS no. 34552–83–5), pentadecanoic acid (CAS no. 1002–84–2), oleic acid (CAS no. 112–80–1), and DC260126 (CAS no. 346692–04–4) were purchased from MedChemExpress (NJ, USA). Fluo-4 AM (S1060) was obtained from Beyotime (Shanghai, China). CMC-Na (CAS no. 9004–32–4) was supplied by Tianjin Zhonglian Chemical Reagent Co., Ltd. (Tianjin, China). Activated charcoal powder (CAS no. 7440–44–0) was obtained from Aladdin (Shanghai, China). SYBR (Q711–02) was purchased from Vazyme Biotech Co., Ltd. (Nanjing, China). To prepare the activated charcoal solution, 5 g of activated charcoal was added to 100 mL of 0.5% aqueous CMC-Na solution. The rhizome of *Polygonatum cyrtonema* Hua was collected from Heping Village, Heping Town, and Shaowu City, Fujian Province, China.

### 2.2 Preparation of PCP

As illustrated in Figure 1A, PCP was extracted and separated from the steamed *P. cyrtonema* Hua rhizome. The powdered rhizomes (100 g) were ultrasonically treated with 1 L of 80% (v/v) EtOH twice for 30 min each. Following spinning, the formed pellet was subjected to polysaccharide extraction: two times using 2 L of water at 90 °C for 1 h each. The combined aqueous extracts were then precipitated with three volumes of 95% (v/v) EtOH. The precipitate was subsequently redissolved in water, purified by dialysis, and finally dried to yield PCP.

**Figure 1:**
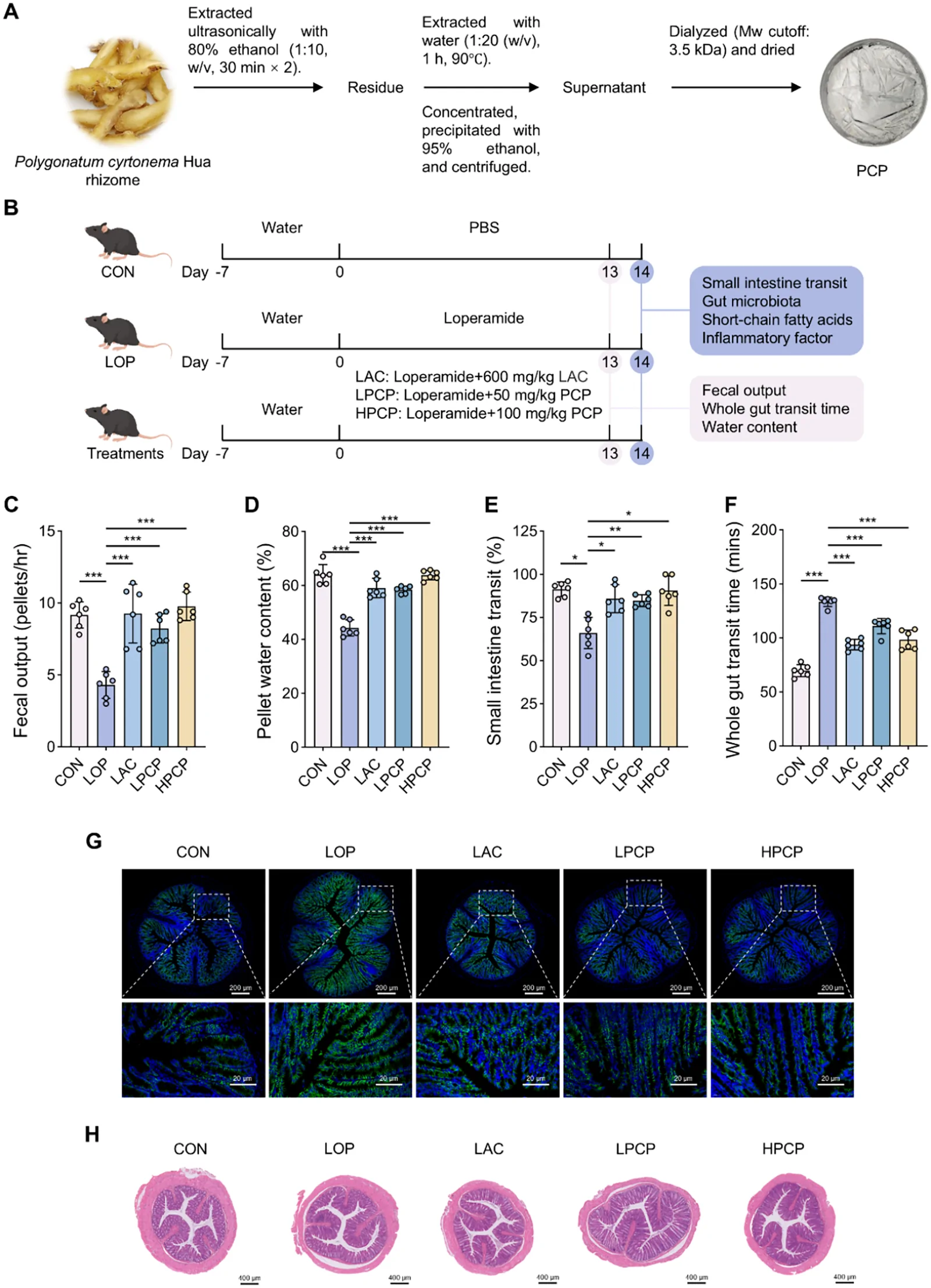
PCP increased fecal output, pellet water content, and promoted colonic motility in constipated mice. (A) Extraction flow of PCP. (B) Schematic illustration of the experiment design. (C) Fecal output. (D) Pellet water content. (E) Small intestine transit rate. (F) Whole gut transit time. (G) Representative IF staining images of AQP4 in colon tissues. Scale bars: 200 μm (upper), 20 μm (lower). (H) Representative images of HE-stained colonic sections. Scale bar: 400 μm. Data represent the mean ± SD (*n* = 6 for (B–F); *n* = 3 for G-H). *P < 0.05, **P < 0.01, and ***P < 0.001.

### 2.3 Physicochemical Identification of PCP

The total polysaccharide content of PCP was determined by the phenol-sulfuric acid method using glucose as the standard. The surface morphology of PCP was characterized by SEM (Hitachi SU8100, Japan) at 3.0 kV; samples were gold-coated and imaged at 200× magnification. The molecular weight was measured by HPSEC-MALLS-RID following the protocol previously established by Siet al.^14^ The monosaccharide composition was measured as described in the assay previously published by Yuan et al.^19^ Fructose content was quantified by HPLC-CAD on a Waters-NH_2_ column. The remaining monosaccharides were measured by PMP precolumn derivatization followed by HPLC-UV on an Agilent Eclipse XDB-C_18_ column.

### 2.4 Animal Experiments

All animal experiments complied with the ARRIVE guidelines and the National Research Council’s Guide for the Care and Use of Laboratory Animals, authorized by Zunyi Medical University (Ethics No.: ZHSC-2-[2024]­101). Male and female C57BL/6 mice at 6 weeks were supplied by Guangdong Zhiyuan Biomedical Technology Co., Ltd. (Guangdong, China) and allowed to acclimate for 7 days. During the experimental period, no significant differences in daily food intake were observed among all groups, with an average consumption of approximately 2.5 g/mouse/day, excluding the possibility that altered food intake contributed to the differences in defecation parameters.

#### 2.4.1 Assessment of PCP Effects

The study was conducted separately for male and female mice. For each sex, mice were randomly separated into five groups (*n* = 6 per group). During day 0 to day 14, the CON group was given phosphate-buffered saline (PBS), while the remaining four groups were administered 10 mg/kg loperamide to induce constipation. At 1 h post-loperamide daily, PBS was given by gavage to the LOP group, whereas the other three treatment groups were orally administered lactulose (600 mg/kg) (LAC group), low-dose PCP (50 mg/kg, LPCP group), and high-dose PCP (100 mg/kg, HPCP group), respectively. Body weight was measured each day. On the last day, blood was drawn from all mice before they were sacrificed by CO_2_ asphyxiation. The small intestine, colon, heart, liver, spleen, lung, kidney, feces, and cecal contents were harvested. 1.0 cm of colon was obtained and immersed in 4% paraformaldehyde for histopathological and immunofluorescence (IF) staining. After letting the blood stand at room temperature for 2 h, the serum was collected by centrifugation at 3000 rpm for 15 min. The residual colon tissue, feces, cecal contents, and serum were quick-frozen and preserved at −80 °C for subsequent measurements.

#### 2.4.2 FMT

The FMT experiment was conducted by the method previously presented by He et al.,^21^ with minor adjustments. To avoid the interference caused by the age mismatch, donor mice aged 4–6 weeks were used, which reached 6–8 weeks by the time of transplantation, matching the age of recipient mice. Male C57BL/6 donor mice were randomly allocated to three different groups of 14 animals each: d-CON, d-LPCP, and d-HPCP groups. d-LPCP and d-HPCP groups of mice were orally administered PCP at doses of 50 mg/kg and 100 mg/kg, respectively; the d-CON group of mice was orally administered PBS for 14 consecutive days. Freshly collected feces were gathered daily from donor mice throughout the treatment period. On each day, pooled fecal samples from each donor group were homogenized in PBS to a 200 mg/mL suspension, and then centrifuged at 100*g* (4 °C, 5 min). The supernatant was then used for transplantation.

Male C57BL/6 recipient mice (6–8 weeks) were randomly allocated to six groups (*n* = 6 per group). The CON group received PBS from day 0 to day 14, whereas the remaining five groups were administered 10 mg/kg loperamide for 14 successive days after a 7-day adaptation period. One hour after each loperamide administration, the designated recipient groups received fecal microbiota from the d-LPCP (FMT-LPCP group), d-HPCP (FMT-HPCP group), and d-CON donors (FMT-CON group), respectively, by intragastric gavage. The LAC group was intragastrically administered lactulose (600 mg/kg), while the LOP group was given PBS in the same manner. At the end of the experiment, all mice were sacrificed by CO_2_ exposure, and the small intestine, colon, feces, and cecal contents were sampled. 1.0 cm of colon was taken and immersed in 4% paraformaldehyde for histopathological and IF staining. The leftover colon tissue, feces, and cecal contents were flash-frozen and kept at −80 °C for later assays.

#### 2.4.3 Assessment of PDA Effects

Male mice were randomly allocated to five groups (*n* = 6 per group). The CON group was given PBS, while the rest of the four groups were administered 10 mg/kg loperamide from day 0 to day 14 to induce constipation. Mice in the LAC group were orally administered lactulose (600 mg/kg). Mice in the LPDA and HPDA groups were administered PDA at doses of 10 mg/kg and 40 mg/kg, respectively. All treatments were administered daily from day 0 to day 14, during which body weight was monitored daily. On day 14, following the final administration, all mice were sacrificed via CO_2_ exposure, and the small intestine, colon, feces, and cecal contents were harvested. A 1.0 cm segment of the colon was fixed in 4% paraformaldehyde for subsequent histopathological and IF staining. The remaining colonic tissues, along with the feces and cecal contents, were quick-frozen and preserved at −80 °C for further assays.

#### 2.4.4 Function of GPR40 in Constipation Relief

Male mice were assigned to four groups (*n* = 6 per group) in a randomized manner: CON, LOP, PDA, and DC260126. The CON group was administered PBS. The remaining three groups (LOP, PDA, and DC260126) were administered loperamide (10 mg/kg) daily for 14 days to induce constipation. In addition, the PDA and DC260126 groups received PDA (40 mg/kg) orally, and the DC260126 group was intraperitoneally injected with DC260126 (20 mg/kg) immediately before each PDA administration. Body weight was documented daily. On day 14, upon completion of the final dosing, all mice were sacrificed by CO_2_ asphyxiation, and the small intestine, colon, feces, and cecal contents were collected, quick-frozen, and maintained at −80 °C for subsequent analysis.

### 2.5 Determination of Fecal Output, Whole Gut Transit Time, and Fecal Pellet Water Content

On day 13, the mice were placed on a 12 h fast and then received activated charcoal powder via gavage. Afterward, every mouse was placed alone in a new cage, and food and water were provided without restriction. For each mouse, the time elapsed from activated charcoal administration to the first emergence of black feces was measured, and the number of fecal pellets produced in 5 h after intragastric administration was quantified. The feces obtained within 5 h were lyophilized, and their water content was determined.

### 2.6 Evaluation of Small Intestine Transit Rate

As in the evaluation of the whole gut transit time, mice underwent a 12 h fasting period before being given activated charcoal powder by oral gavage. Twenty minutes later, the mice were sacrificed, and the entire small intestine was removed via abdominal dissection. Both the total length of the small intestine and the distance traveled by the carbon powder were documented, and the small-intestinal transit rate was subsequently computed.

### 2.7 Hematoxylin-Eosin Staining

Mouse colons were subjected to fixation with 4% paraformaldehyde. After 48 h, the fixed tissues were removed and flushed with a continuous stream of water for 4 h. The samples were then mounted in paraffin, sectioned into 5 μm slices, and the slices were transferred to a tepid water trough to flatten. Subsequently, the samples were stained with Hematoxylin-Eosin (H&E) (Sigma-Aldrich Co.). The morphological details were visualized using an optical microscope. The tissues were inspected at 400× magnification, and images were taken (Leica, Germany).

### 2.8 Indirect Immunofluorescence Assay

Following tissue collection and paraffin embedding after dehydration, Aquaporin 4 (AQP4) was assessed using immunofluorescence staining. Tissue sections (5 μm thick) were dewaxed and rehydrated following routine procedures, then blocked in PBS containing 3% goat serum for 30 min at 37 °C to block nonspecific binding. Subsequently, the sections were treated with primary antibody against AQP4 (rabbit, Abcam, 1:500) overnight at 4 °C. This was followed by incubation with the secondary antibody Cy3 (anti-rabbit, Jackson, 1:200) for 30 min at 37 °C. A negative control was prepared by excluding the primary antibody. Cell nuclei were labeled with DAPI for visualization.

### 2.9 Gut Microbiota Analysis

Microbial genomic DNA was extracted from mouse fecal samples. The V3–V4 region of the bacterial 16S rRNA gene was amplified, and the purified amplicons were subsequently sequenced using the Illumina MiSeq platform. α- and β-diversity analysis and visualizations, including Venn diagrams, pie charts, bar plots, and hierarchical clustering heatmaps, were generated using the Majorbio Cloud Platform (www.majorbio.com) to compare gut microbiota composition among the five experimental groups.

### 2.10 Nontargeted Metabolomics Analysis

Untargeted fecal metabolomics analysis was conducted using LC-MS at Shanghai MajorBio Bio-Pharm Technology Co., Ltd. (Shanghai, China). In brief, 50 mg of feces and 400 μL of 80% methanol extraction solvent (containing l-2-chlorophenylalanine as an internal standard at 0.02 mg/mL) were milled for 5 min, followed by extraction via sonication for 20 min (4 °C, 40 kHz). After centrifugation, the supernatant was subjected to LC-MS analysis. A UPLC-Q-Exactive Orbitrap mass spectrometer (Thermo Fisher Scientific) equipped with a BEH C18 column (100 mm × 2.1 mm, 1.7 μm; Waters) was employed. All samples were analyzed in both positive and negative ion modes, and data-dependent MS/MS spectra were acquired over an *m*/*z* range of 81–1000. PCA and PLS-DA were performed using R, and metabolites with VIP values >1 and P-values <0.05 were considered significant.

### 2.11 SCFA Detection

Measure 20 mg of cecum contents, mix with 800 μL of 0.5% phosphoric acid (aq, containing the reference standard 2-ethylbutyric acid at 10 μg/mL), homogenize (180 s, 50 Hz), ultrasonicate for 10 min, and centrifuge (4 °C, 15 min, 13,000 rcf) to get the supernatant. Add 200 μL of *n*-butanol for solvent extraction, mix for 10 s, ultrasonicate for 10 min, and centrifuge (4 °C, 5 min, 13,000 rcf) to get supernatant. The suspension was then passed through a 0.22 μm organic filter membrane, and thereafter quantified by GC (30 m × 0.25 mm × 0.25 μm, 6890 N, Agilent, USA) with an Electron impact ion source (EI). The carrier gas was helium (1.0 mL/min), and a sample of 1.0 μL was loaded for analysis. The temperature gradient for the oven was set as follows: the starting temperature was established at 80 °C, followed by programmed heating at a rate of 20 °C/min to 120 °C, then at 5 °C/min to 160 °C, and finally held at 220 °C for 3 min.

### 2.12 Medium- and Long-Chain Fatty Acid Detection

Approximately 50 mg of cecal contents were accurately weighed, thereafter homogenized with 1 mL of CH_2_Cl_2_: MeOH (v/v = 1:1), subjected to 50 Hz agitation for 3 min. The mixture was then treated with ultrasound for 15 min and left to stand at −20 °C for 15 min. After spinning at 4 °C and 13,000 rcf for 10 min, the supernatant was recovered and dried via a nitrogen stream. After that, 0.5 mL of NaOH (MeOH solution, 0.5 mol/L) was introduced, followed by vortexing for 30 s and incubation at 60 °C for 0.5 h. Once cooled, 0.5 mL of *n*-hexane was supplemented, and the mixture was agitated for 30 s, spun at 4 °C and 13,000 rcf for 10 min. Then, 200 μL of the *n*-hexane layer was loaded into an injection vial for GC-MS analysis. Targeted metabolomic analysis was undertaken with an Agilent 8890B-5977*B*/7000D GC MS system equipped with an EI source. Helium was supplied as the carrier gas at a constant flow rate of 1.0 mL/min, and the injector was maintained at 230 °C. Each sample was injected at a volume of 1 μL. We programmed the column temperature as follows: starting at 80 °C with no hold, heating to 180 °C at 8 °C/min, then to 220 °C at 4 °C/min, then to 230 °C at 2 °C/min, and holding at 230 °C for 13.5 min. A postrun step at 235 °C for 2 min was then performed.

### 2.13 *In Vitro* Fermentation of PCP

The BCM contained per liter: peptone, 2 g; yeast extract, 1 g; bile salts, 0.5 g; Tween 80, 2 mL; l-cysteine–HCl, 0.1 g; hemin, 0.001 g; vitamin K1, 0.002 mL; NaCl, 0.1 g; K_2_HPO_4_, 0.04 g; KH_2_PO_4_, 0.04 g; MgSO_4_·7H_2_O, 0.01 g; CaCl_2_·2H_2_O, 0.01 g; NaHCO_3_, 0.2 g; and resazurin, 0.0005 g (pH 6.0). The medium was boiled, autoclaved at 120 °C for 15 min, and stored at 4 °C under anaerobic conditions. Polysaccharide samples were dissolved in BCM at 6 mg/mL. Glucose and blank (BCM only) served as positive and negative controls, respectively.

Fresh feces from constipated mice were homogenized in 20 mL of PBS (containing 0.5 g/L l-cysteine–HCl) for 5 min. The homogenate was centrifuged at 500*g* for 1 min to remove debris, and the supernatant was centrifuged at 8000*g* for 10 min to collect bacterial pellets, which were resuspended in 12.5 mL of anaerobic PBS (with 0.5 g/L l-cysteine–HCl). Then, 1 mL of the bacterial suspension was inoculated into 9 mL of BCM (with or without carbohydrates) in sealed serum bottles.

Anaerobic fermentation was conducted at 37 °C for 72 h in an anaerobic workstation (LAI-3T-NDF-011, Shanghai Longyue, China). Samples were collected at 0, 12, 24, 48, and 72 h and stored at −80 °C. At 72 h, 9 mL aliquots were centrifuged at 8000 *g* for 10 min at 4 °C. The supernatants were used for indicator assays, and the pellets were used for bacterial DNA extraction.

### 2.14 Quantitative PCR Analyses

DNA was extracted from fermentation samples with the Bacterial Genomic DNA Extraction Kit (DP302–02, Tiangen). *Lachnospiraceae*-specific primers (forward, 5′-CGGTACCTGACTAAGAAGC-3′; reverse, 5′-AGTTT­(C/T)­ATTCTTGCGAACG-3′) and universal 16S rRNA primers (forward, 5′-ACTCCTACGGGAGGCAGCAG-3′; reverse, 5′-ATTACCGCGGCTGCTGG-3′) were used for qPCR amplification on an ABI QS7 Flex system (Thermo Fisher, USA) with SYBR Green. Cycling conditions were 95 °C for 30 s, 40 cycles of 95 °C for 10 s, 60 °C for 30 s, and 72 °C for 30 s. Relative abundance of *Lachnospiraceae* was calculated using the 2^–ΔCT^ method, with the universal 16S rRNA gene as the ref 22.

### 2.15 Molecular Docking

Molecular docking was performed with AutoDock v4.2.6 to explore the interaction between the isolated compounds and the GPR40 receptor (PDB: 4PHU). Ligand geometries were energy-minimized before docking to avoid structural distortions. AutoDock’s scoring function was applied to assess binding affinities, and the resultant docking conformations were displayed using PyMOL 3.1.6.1 and Ligplot+ v.2.2.4.

### 2.16 Cell Culture

Human intestinal Caco-2 cells were obtained from American Type Culture Collection (ATCC, Manassas, VA) and cultured in T-25 flasks in RPMI1640 medium (Gibco) supplemented with penicillin (100 units/mL), streptomycin (100 μg/mL), and fetal bovine serum (15%). Cells were incubated at 37 °C with 5% CO_2_. The culture medium was changed every 2–3 days, and cells were passaged when confluency reached about 80%–90%.

### 2.17 Intracellular Ca^2+^ Assay

Intracellular calcium levels were measured using the fluorescent indicator Fluo-4 AM. Caco-2 cells were seeded into black 96-well plates at a density of 5 × 10^3^ cells per well and cultured for 24 h. After removal of the culture medium, cells were washed once with PBS and then loaded with 5 μM Fluo-4 AM in HBSS at 37 °C for 30 min in the dark. Following incubation, cells were washed three times with HBSS to remove extracellular dye. For GPR40 agonist assays, cells were treated with various concentrations of PDA or oleic acid (OA) as a positive control. For GPR40 antagonism assays, cells were preincubated with 10 μM DC260126 for 15 min prior to PDA stimulation. Fluorescence intensity was immediately recorded using a microplate reader at excitation/emission wavelengths of 490/525 nm, and intracellular Ca^2+^ levels were expressed as relative fluorescence units.

### 2.18 Statistical Analysis

GraphPad Prism 10 was used for data analysis and graph generation. All results are presented as mean ± standard deviation (SD). Intergroup statistical differences were determined by one-way ANOVA with Tukey’s post hoc test. Comparisons between two groups were evaluated using an unpaired *t*-test.

## 3 Results

### 3.1 Physicochemical Identification of PCP

The total polysaccharide content of PCP was 88.6%, confirming a relatively high purity of the extracted polysaccharide. To verify the structural consistency of the extracted PCP, the physicochemical profiles were characterized (Figure S1). SEM analysis revealed that PCP exhibited a sheet-like surface morphology with irregular voids (Figure S1A). GPC/SEC data demonstrated that PCP had an absolute molecular weight of 1.78 × 10^4^ Da (Figure S1B) and was predominantly composed of fructose (77.4%), mannose (7.4%), and glucose (7.5%) (Figure S1C–D). These physicochemical characteristics were consistent with those of the PCP previously characterized by methylation-GC-MS and 1D/2D NMR, which was identified as an inulin neoseries-type fructan composed predominantly of β-(2→1)-linked fructofuranosyl units.^19^ The consistency in molecular weight, monosaccharide composition, and surface morphology between the present and previously characterized batches supports the reproducibility of the PCP preparation used in this study. However, full structural re-elucidation of the present batch was not repeated, and the structural assignment relies on the previously established structure of PCP together with the present physicochemical characterization.

### 3.2 PCP Ameliorated Constipation in Mice

To evaluate the mitigative effect of PCP on constipation, loperamide-induced constipated mice were treated with 50 or 100 mg/kg PCP (LPCP or HPCP group). Simultaneously, a positive control group received 600 mg/kg BW lactulose (LAC group), a well-established anticonstipation agent (Figure 1B). Loperamide successfully caused constipation, as indicated by markedly reduced fecal output (Figure 1C) and pellet water content (Figure 1D), decreased small intestine transit rate (Figure 1E), and prolonged whole gut transit time (Figure 1F). High-dose PCP (HPCP group) significantly increased fecal output (Figure 1C) and pellet water content to approximately 64%, analogous to the CON group, and lactulose-treated mice displayed comparable results (Figure 1C,D). The high-dose PCP and lactulose raised the small intestine transit rate to approximately 90% and 86%, respectively (Figure 1E). Furthermore, the high-dose PCP reduced whole gut transit time to approximately 97 min, which was comparable to the LAC group (approximately 96 min, Figure 1F). AQPs are water channels that selectively transport water and glycerol and play key roles in the body’s water transport system.^23^ AQP4 resides in the basolateral membrane of colonic epithelial cells,^24^ facilitates water transport from the luminal side to the vascular side when its expression levels increase in the colon, thereby hardening stools and causing constipation.^25^ Immunofluorescence staining revealed that AQP4 expression in the colon was markedly elevated in the LOP group compared to the CON group. However, both doses of PCP significantly downregulated AQP4 expression relative to that in the LOP group (Figure 1G), and none of the treatments caused obvious gut damage (Figure 1H). In contrast, AQP3 expression showed no significant change among all groups (Figure S2). The experimental results in female mice were consistent with those in male mice (Figure S3). For subsequent mechanistic experiments, male mice were used to minimize potential confounding effects related to ovarian hormone fluctuations and the estrous cycle, which may influence gastrointestinal motility, intestinal transit, and microbiota-associated phenotypes.

These findings underscore PCP’s efficacy in mitigating constipation, achieved through the dual mechanisms of downregulating colonic AQP4 expression to increase pellet water content and enhancing colonic motility.

### 3.3 PCP Ameliorated Gut Microbiota Dysbiosis in Constipated Mice

To explore the possibility that PCP could mitigate constipation-induced microbiota imbalance, we profiled the gut microbiota via 16S rRNA gene sequencing. Venn diagrams revealed that 1308, 897, 1626, 961, and 1075 distinct amplicon sequence variants (ASVs) were identified in CON, LOP, LAC, LPCP, and HPCP groups, respectively (Figure 2A). α diversity analyses, including Shannon, Ace, Chao, Sobs, Simpson, and Coverage indices, failed to detect significant differences among the five groups (Figure S4A–F), showing that PCP and lactulose did not affect bacterial richness or diversity. Nonetheless, a comparison at the ASV level indicated that both doses of PCP reshaped the microbiota composition in constipated mice. To assess β diversity, the overall structure of the gut microbiota was illustrated by principal coordinate analysis (PCoA) (Figure 2B) and nonmetric multidimensional scaling (NMDS) (Figure S4I). Significant separation was observed between the CON and LOP groups, and the PCP-treated groups (LPCP and HPCP) clustered separately from the LOP group, suggesting that PCP reshaped the overall gut microbial structure (Figures 2B, S4I).

**Figure 2:**
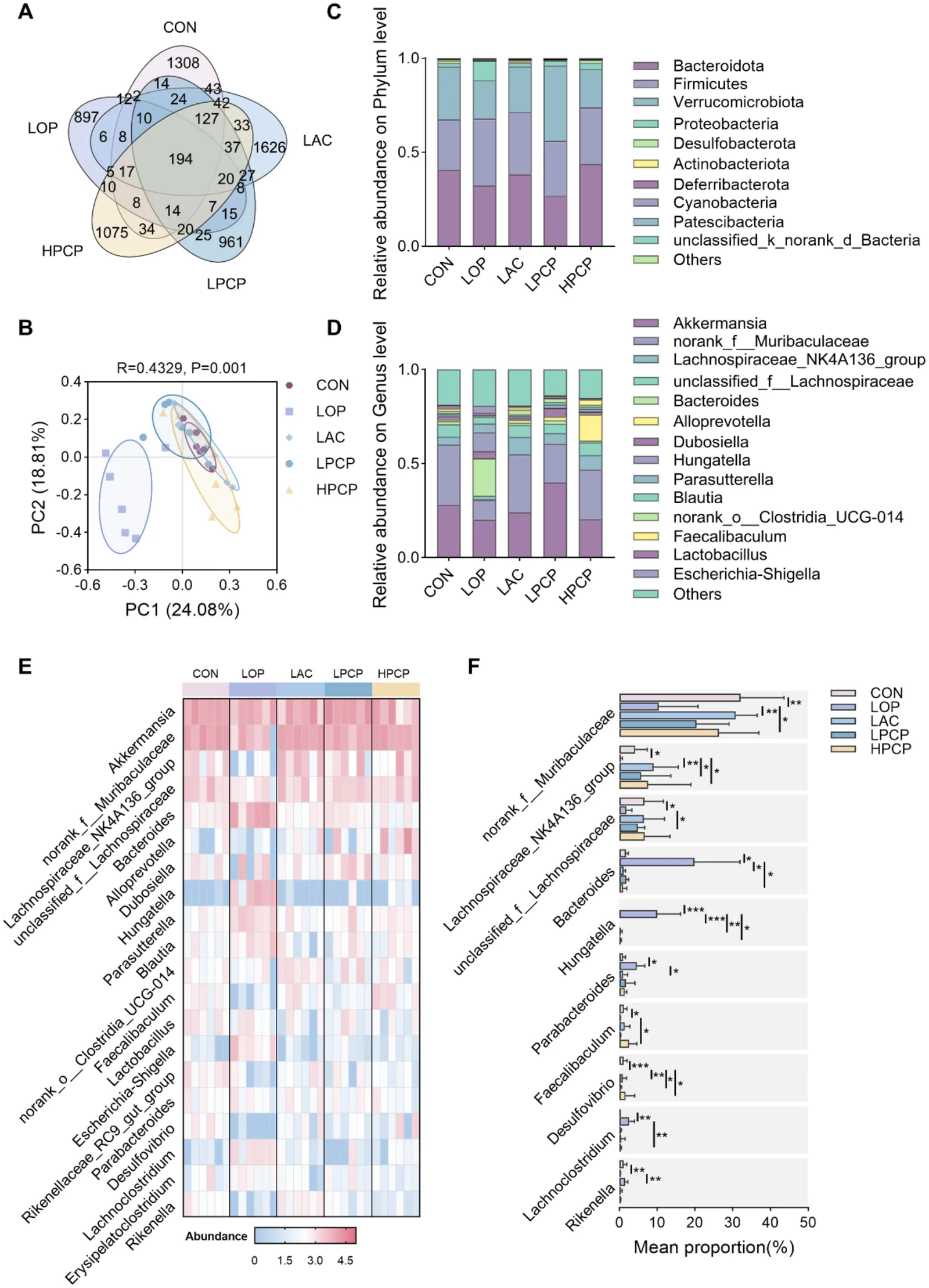
PCP ameliorated gut microbiota dysbiosis in constipated mice. (A) Petal Venn diagram of gut microbiota sets between different groups. Numbers in the Venn diagram indicate the number of ASVs in each group (petals) and shared ASVs among groups (center). (B) PCoA analysis at the ASV level. (C, D) Relative abundance of the gut microbiota at phylum (C) and genus (D) levels. (E) Heatmap of the relative abundance of the top 20 genera. (F) Top 10 differential species in relative abundance. Data represent the mean ± SD (*n* = 6). *P < 0.05, **P < 0.01, and ***P < 0.001.

At the phylum level (Figure 2C), the LOP group showed an enrichment of *Proteobacteria* compared with the CON group. The LPCP group showed an enrichment of *Verrucomicrobiota*, and the HPCP group exhibited an increased relative abundance of *Bacteroidota* relative to the LOP group. At the genus level, PCP intervention remarkably elevated the relative abundances of potentially beneficial bacteria, including *norank_f__Muribaculaceae*, *Lachnospiraceae_NK4A136_group*, *unclassified_f__Lachnospiraceae* (Figure 2D–**F**), *norank_f__UCG 010* (Figure S4G), and *Muribaculum* (Figure S4H). In contrast, constipation resulted in a substantial surge in the abundance of potentially harmful bacteria *Hungatella* and *Parasutterella* (Figure 2D–F), which was dramatically reversed by PCP treatment. Therefore, PCP alleviated constipation-induced microbiota dysbiosis.

Collectively, these findings indicate that PCP treatment not only remodeled the gut microbiota composition in constipated mice but also alleviated constipation-induced gut microbiota dysbiosis.

### 3.4 FMT Relieved the Symptoms of Constipation

There is a broad consensus that gut microbiota and their bioactive metabolites play essential roles in the pathogenesis of constipation. After confirming that PCP alleviates constipation-induced gut microbiota dysbiosis, we further investigated whether the gut microbiota serves as a key mediator of the alleviation of constipation by PCP. As demonstrated in Figure 3A, feces from PCP-treated donor mice (d-LPCP and d-HPCP groups, respectively) or normal donor mice were harvested, and the fecal microbiota was processed and administered to constipated recipients (FMT-CON, FMT-LPCP, and FMT-HPCP groups, respectively) by gavage. Following FMT, constipated recipients (FMT-LPCP and FMT-HPCP groups) who received microbiota from PCP-treated donors showed a significant increase in fecal output (Figure 3B), pellet water content (Figure 3C), and small intestine transit rate (Figure 3D), as well as a pronounced reduction in whole gut transit time (Figure 3E) and downregulated AQP4 expression (Figure 3F). This therapeutic effect was also significantly superior to that of the FMT-CON group, indicating that the efficacy originated from the altered gut microbiota of donor mice following PCP treatment, rather than from the gut microbiota of normal mice (Figure 3B–E). Consequently, FMT from PCP-treated mice markedly ameliorated loperamide-induced constipation.

**Figure 3:**
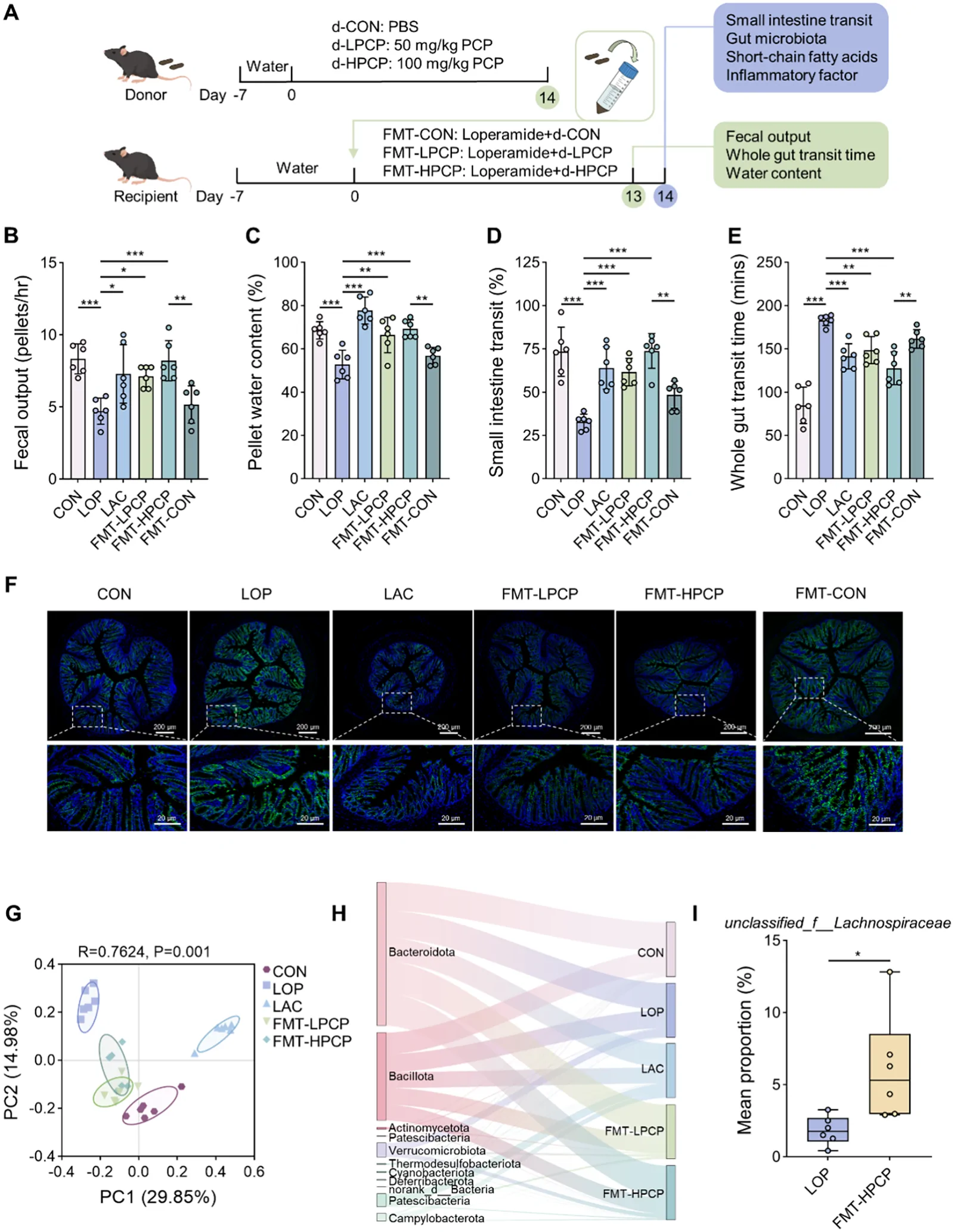
Effect of FMT on constipation-related phenotypes and gut microbiota composition in mice. (A) Schematic illustration of the animal experiment design. (B) Fecal output. (C) Pellet water content. (D) Small intestine transit rate. (E) Whole gut transit time. (F) Representative IF staining images of AQP4 in colon tissues. Scale bars: 200 μm (upper), 20 μm (lower). (G). PCoA analysis at the ASV level. (H) Relative phylum abundance of the gut microbiota. (I) Relative abundance of *unclassified_f__Lachnospiraceae*. Data represent the mean ± SD *n* = 6 for (B–E, G–I); *n* = 3 for (F). *P < 0.05, **P < 0.01, and ***P < 0.001.

Moreover, 16S rRNA gene sequencing analysis of the recipients’ feces displayed that the gut microbial structure of the FMT-LPCP and FMT-HPCP groups clustered closely with that of their respective PCP-treated donors. There were no notable distinctions in the α-diversity of the gut microbiota among groups of constipated mice following FMT treatment. PCoA and NMDS at the ASV level were used to assess the distinctions in the composition of the mice colonies in each group. PCoA (Figure 3G) and NMDS (Figure S5I) showed that the LOP group was different from the CON group. Meanwhile, the distance between the CON and the FMT-treated groups was smaller than the spacing between the CON and the LOP group, which indicated that FMT treatment also ameliorated the gut microbiota dysbiosis in loperamide-induced constipated mice. Consistent with the HPCP group, at the phylum level (Figure 3H), the FMT-HPCP group exhibited a higher relative abundance of *Bacteroidota* compared with the LOP group. At the genus level (Figure S5J), PCP administration significantly increased the relative abundances of *unclassified_f__Lachnospiraceae* (Figure 3I), *norank_f__UCG 010* (Figure S5G), and *Muribaculum* (Figure S5H). In both pharmacodynamic and FMT experiments, *unclassified_f__Lachnospiraceae* maintained a high relative abundance.

On the whole, the gut microbiota is proposed as a critical mediator in the alleviation of loperamide-induced constipation by the PCP. In this process, *unclassified_f__Lachnospiraceae* may represent an important functional bacterial genus.

### 3.5 PCP Enhances Gut Microbiota-Derived Metabolite Production

In light of the crucial role of gut microbiota–host metabolic interactions, as well as the significance of microbiota-derived metabolites in constipation, we performed untargeted metabolomic analysis on fecal samples previously subjected to 16S rRNA gene sequencing. To assess metabolic differences among the five groups, we conducted principal component analysis (PCA) (Figure 4A) and partial least-squares-discriminant analysis (PLS-DA) (Figure S6A). The score plots revealed clear separation among the CON, LOP, LAC, LPCP, and HPCP groups. Notably, the PCP-treated groups clustered more closely with the CON group than with the LOP group, indicating that PCP effectively alleviated the constipation-induced metabolic disturbance (Figures 4A, S6A).

**Figure 4:**
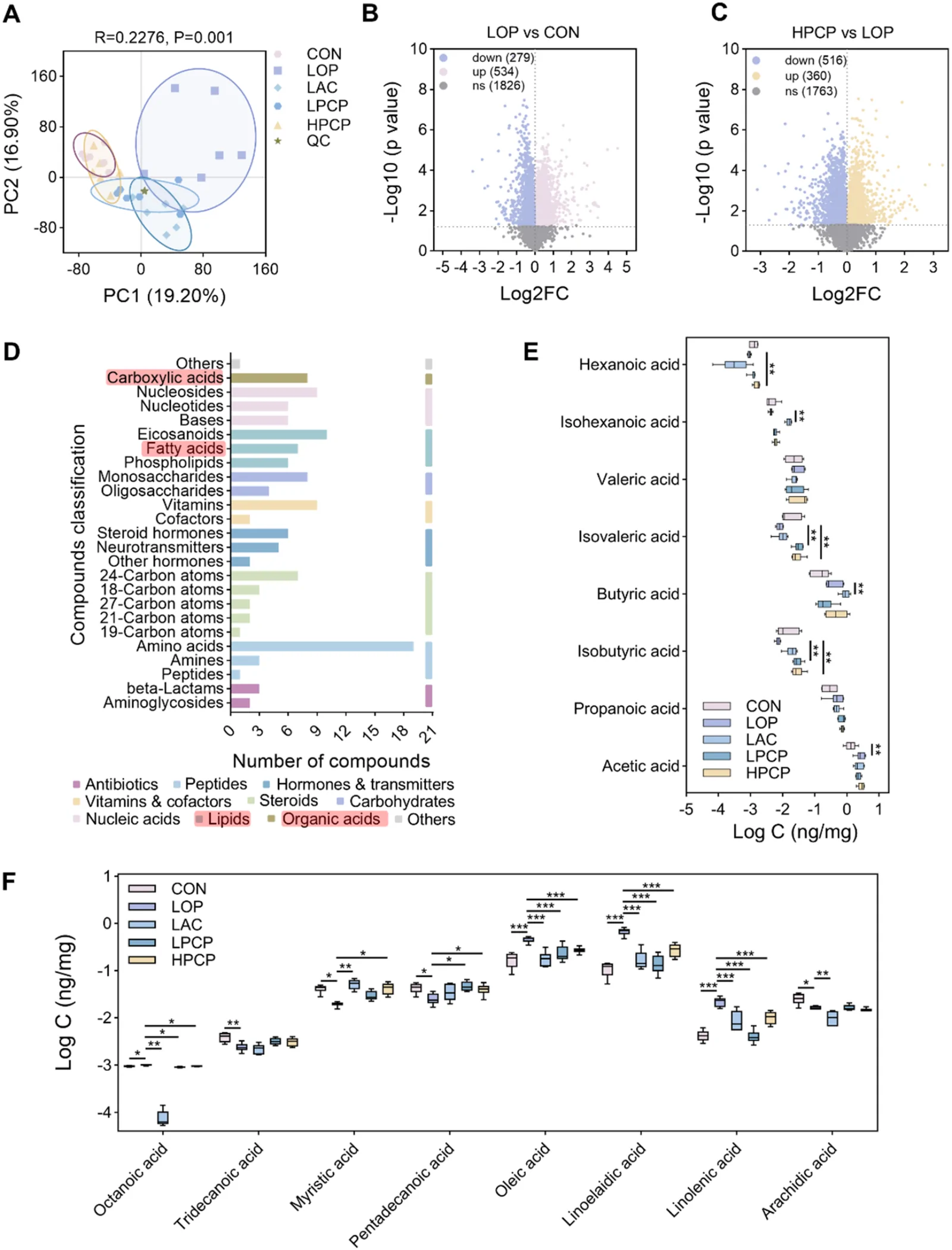
Effect of PCP on metabolomic profiles in constipated mice. (A) PCA score plot of metabolomic features among groups. (B) Volcano plot of differential metabolites between CON and LOP groups. (C) Volcano plot of differential metabolites between HPCP and LOP groups. (D) KEGG compound classification of differential metabolites. (E) Quantification of SCFAs in mouse cecal contents. (F) Quantification of MLCFAs in mouse cecal contents. Data represent the mean ± SD (*n* = 6). *P < 0.05, **P < 0.01, and ***P < 0.001.

Overall, 2639 differentially abundant metabolites were characterized across all of the comparisons. The volcano plot analysis demonstrated that compared with the CON group, 279 metabolites were upregulated and 534 were downregulated in the LOP group (Figures 4B and S6C). In contrast, 360 metabolites were increased and 516 were decreased in the HPCP group in comparison with the LOP group in colonic contents (Figures 4C and S6C). Classification analysis based on KEGG (Figure 4D) and HMDB (Figure S6D) databases indicated that amino acids, organic acids, and lipids represented the major classes of differential compounds. KEGG pathway enrichment further highlighted amino acid metabolism and aldosterone-related pathways as the most significantly altered metabolic routes (Figure S6E).

On the basis of the above findings, targeted metabolomic analysis was performed to quantify SCFAs (Figure 4E) and medium- to long-chain fatty acids (MLCFAs) (Figures 4F, S6F). The results showed that PCP intervention at different doses markedly increased the concentrations of the branched-chain SCFAs isobutyric acid, isovaleric acid, and isohexanoic acid (Figure 4E). Regarding MLCFAs, PCP administration significantly increased intestinal levels of octanoic acid, myristic acid, and PDA, while suppressing the production of elaidic acid, linoelaidic acid, and linolenic acid (Figure 4F). Furthermore, no significant difference in the PDA level was observed between the lactulose-treated group and the constipated model group, but the levels of butyric acid, isohexanoic acid, and myristic acid were markedly increased.

Collectively, these results demonstrated that PCP enhanced the production of gut microbiota-derived metabolites, particularly organic acids, suggesting that these metabolites may contribute to the protective effect of PCP against loperamide-induced constipation.

### 3.6 Multiomics Integration of Host–Microbiome–Metabolite Connections

To elucidate the contribution of the gut microbiome to host phenotypes, a joint Spearman correlation analysis was performed between the gut microbiota and the phenotypic parameters (Figure 5A). The results showed that *Bacteroides*, *Hungatella*, *Parasutterella*, and *Escherichia*–*Shigella* exhibited a positive correlation with whole gut transit time, but a negative correlation was observed with fecal output, pellet water content, and small intestine transit. Furthermore, we found that *norank_f__Muribaculaceae*, *unclassified_f__Lachnospiraceae*, *Faecalibaculum*, *Desulfovibrio*, and *Lachnospiraceae_NK4A136_group* were positively correlated with fecal output, pellet water content, and small intestine transit while showing a negative correlation with whole gut transit time. Among them, *unclassified_f__Lachnospiraceae* and *Desulfovibrio* showed the strongest correlations with the host phenotypes. Both the FMT experiment and the correlation analysis indicated that *unclassified_f__Lachnospiraceae* was a key microorganism influencing the host phenotypes.

**Figure 5:**
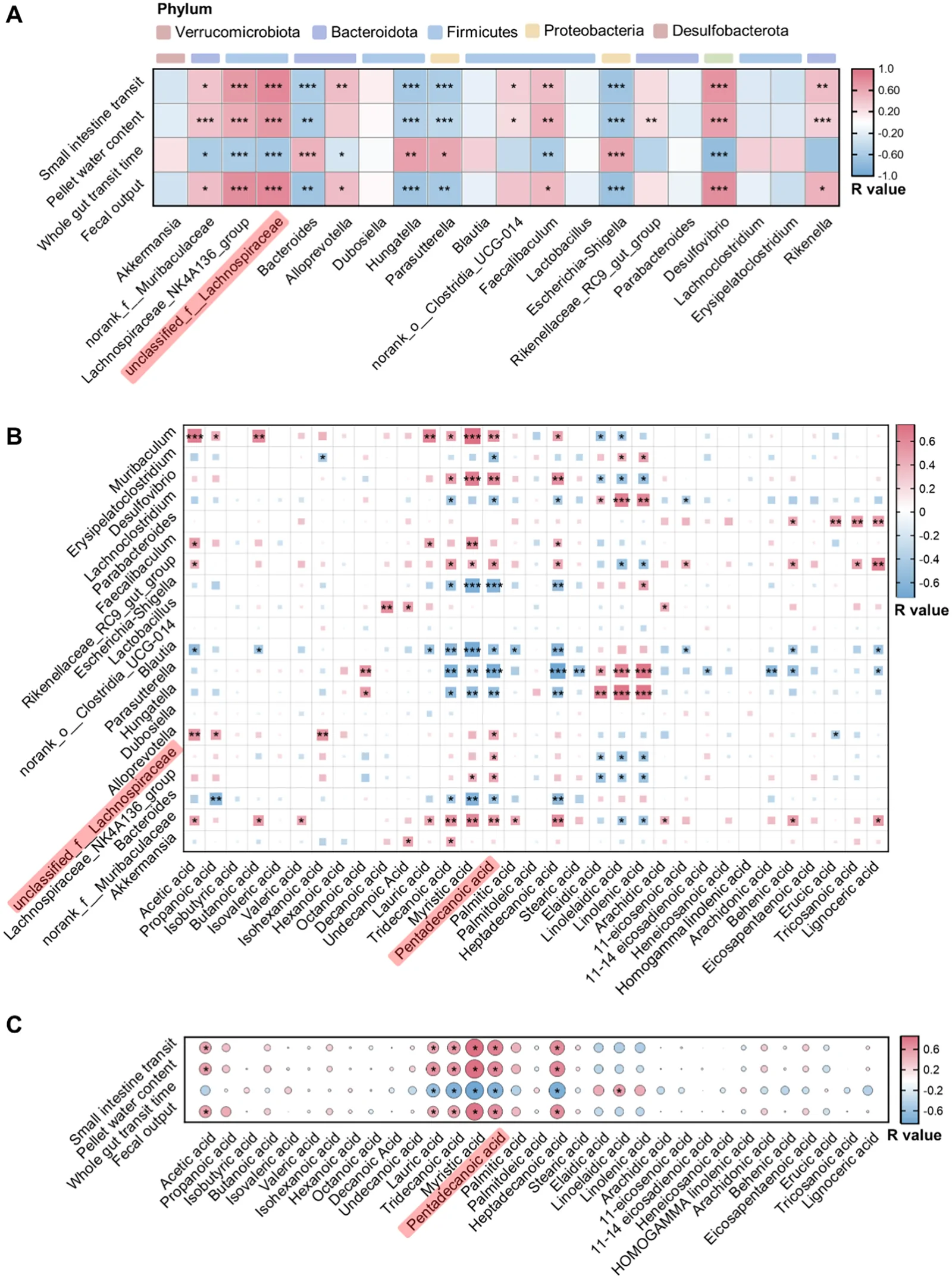
Multiomics integration analysis. (A–C) Heatmaps showing Spearman correlations between host phenotypes and gut microbiota (A), between gut microbiota and SCFAs/MLCFAs (B), and between host phenotypes and SCFAs/MLCFAs (C). *P < 0.05, **P < 0.01, and ***P < 0.001.

To elucidate the interaction more clearly between the gut microbiome and organic acids, we performed Spearman’s correlation analysis between differential gut microbiota and SCFAs and MLCFAs (Figure 5B). The results showed that metabolites associated with potential probiotics were mainly tridecanoic acid, myristic acid, and PDA, whereas those linked to potential pathogens were primarily elaidic acid, linoelaidic acid, and linolenic acid. Among them, the metabolite most significantly positively correlated with *unclassified_f__Lachnospiraceae* was PDA. Additionally, *norank_f__Muribaculaceae*, *Lachnospiraceae_NK4A136_group*, and *Alloprevotella* were also pronouncedly positively correlated with PDA. Previous studies have shown that gut microbiota serve as one of the important producers of odd-chain saturated fatty acids in the host.^15,26–30^ Another study revealed that *Lachnospiraceae* is among the primary producers of PDA.^31^ Therefore, PDA is a microbially derived metabolite rather than an endogenous product.

SCFAs and MLCFAs were also correlated with host phenotypes (Figure 5C). Although the role of SCFAs in alleviating constipation has been widely reported, differential SCFAs showed weak correlations with host phenotypes in the present study, with only acetic acid exhibiting a partial correlation. In contrast, MLCFAs demonstrated a strong correlation. Specifically, PDA was negatively correlated with the whole gut transit time and positively correlated with fecal output, pellet water content, and small intestine transit.

These findings indicate that administration of PCP enhanced microbiota-derived PDA production and that PDA may mediate the ameliorative effect of PCP on loperamide-induced constipation.

*In vitro* fermentation demonstrated that gut microbiota efficiently utilized PCP, as reflected by enhanced bacterial proliferation (Figure S7A) and carbohydrate consumption (Figure S7B). PCP fermentation was accompanied by an increased relative abundance of *Lachnospiraceae* and detectable PDA production (Figure S7C–E). This strongly suggests that *Lachnospiraceae* are key contributors to the conversion of PCP into PDA, reinforcing our *in vivo* findings.

### 3.7 PDA Alleviated Loperamide-Induced Constipation in Mice

To verify the effects of PDA on constipation, loperamide-induced constipated mice were administered 10 mg/kg PDA (LPDA group) or 40 mg/kg PDA (HPDA group). As shown in Figure 6, the fecal output (Figure 6B), the pellet water content (Figure 6C), and the small intestine transit rate (Figure 6D) of loperamide-treated mice (LOP group) were substantially lowered as opposed to the CON group, and the whole gut transit time was substantially lengthened (Figure 6E). Nonetheless, compared with the LOP group, all tested doses of PDA significantly increased the fecal output (Figure 6B), the pellet water content (Figure 6C), and the small intestine transit rate (Figure 6D) and significantly shortened the whole gut transit time (Figure 6E). The LOP group exhibited significantly higher colonic AQP4 expression compared to the CON group. However, treatment with both doses of PDA significantly suppressed this upregulation, and the effect of the HPDA group was more obvious (Figure 6F). These findings substantiate that PDA mediates the alleviating effect of PCP on constipation.

**Figure 6:**
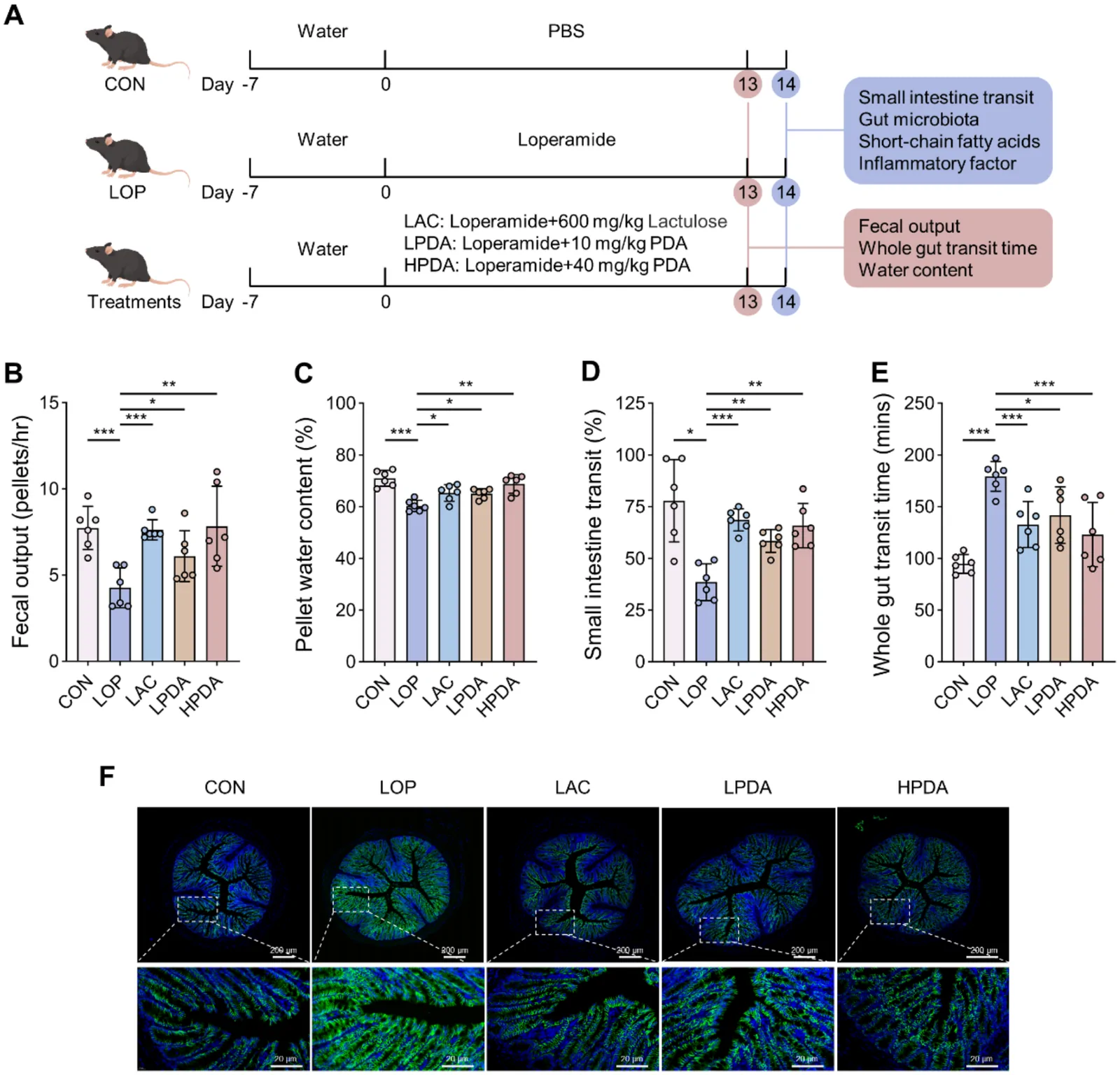
PDA increased fecal output, pellet water content, and promoted colonic motility in constipated mice. (A) Schematic illustration of the animal experiment design. (B) Fecal output. (C) Pellet water content. (D) Small intestine transit rate. (E) Whole gut transit time. (F) Representative IF staining images of AQP4 in colon tissues. Scale bars: 200 μm (upper), 20 μm (lower). Data represent the mean ± SD *n* = 6 for (B–E); *n* = 3 for (F). *P < 0.05, **P < 0.01, and ***P < 0.001.

### 3.8 GPR40 was Essential for PDA to Ameliorate Loperamide-Induced Constipation in Mice

GPR40 is a G-protein-coupled receptor chiefly present in pancreatic islets and the intestine.^32^ It functions as a receptor for MLCFAs.^14^ It has been reported that MLCFAs stimulate colonic motility potentially via enhancement of GPR40-dependent Ca^2+^ influx.^14,15^ In order to explore the agonistic activity of PDA to GPR40, AutoDock software was used for docking. PDA showed a favorable predicted interaction with GPR40, with a calculated binding energy of −7.86 kcal/mol. Lys-259 and Lys-62 form three hydrogen bonds with the carboxyl group. The fatty chain is located inside the hydrophobic pocket formed by Phe-87, Cys-170, Ala-83, Trp-72, Leu-61, Glu-65, Ala-66, Leu-262, and Glu-172 (Figure 7A).

**Figure 7:**
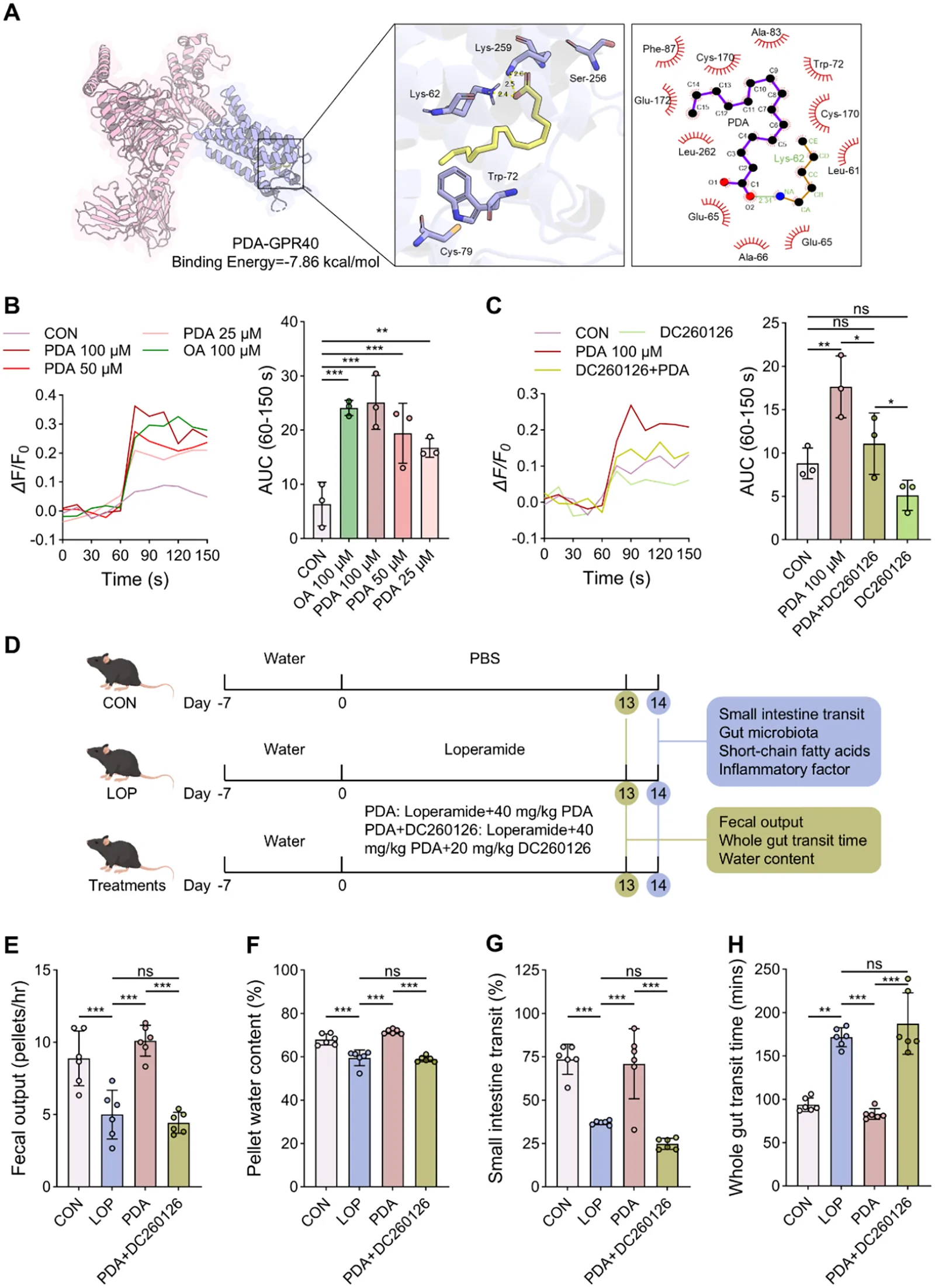
GPR40 antagonist DC260126 reversed the ameliorative effects of PDA on constipation. (A) The binding mode (left), enlarged views of the optimal binding conformation (middle), and 2D molecular docking model (right). (B) PDA treatment caused a concentration-dependent increase in intracellular Ca^2+^ influx (left). The corresponding AUC (60–150 s) bar chart (right) shows significant, concentration-dependent increases. Oleic acid (OA) served as a positive control. (C) Pretreatment with DC260126 specifically inhibited PDA-induced Ca^2+^ influx (left), with the AUC values showing significant reduction (right). (D) Schematic illustration of the animal experiment design. (E) Fecal output. (F) Pellet water content. (G) Small intestine transit rate. (H) Whole gut transit time. Data are presented as the mean ± SD *n* = 3 for (B, C); *n* = 6 for (D–H). *P < 0.05, **P < 0.01, and ***P < 0.001; ns: not significant.

Using the Caco-2 enterocytic line, intracellular Ca^2+^ levels were measured using the Fluo-4 AM fluorescent probe. It was observed that PDA induced a concentration-dependent increase in intracellular calcium concentrations (Figure 7B). Upon preincubation with the GPR40 antagonist DC260126 (10 μM), the intracellular calcium signal induced by 100 μM PDA was diminished (Figure 7C). These findings provide cell-based functional evidence supporting the involvement of GPR40 in PDA-induced Ca^2+^ signaling.

To further investigate the role of GPR40, the GPR40 antagonist DC260126 (20 mg/kg) was administered intraperitoneally before PDA treatment, and host phenotypes were subsequently observed (Figure 7D). Interestingly, treatment with DC260126 abrogated the prokinetic effects of PDA on colon peristalsis in loperamide-induced constipated mice (Figure 7E–H).

In summary, molecular docking results demonstrated that PDA binds favorably to the GPR40. *In vitro* studies showed that PDA activates GPR40 and induces a Ca^2+^ influx in Caco-2 cells. In a loperamide-induced constipation mouse model, the administration of the GPR40 antagonist DC260126 prior to PDA treatment reversed the prokinetic effects of PDA on colon peristalsis. Collectively, these findings suggest that GPR40 may serve as a key mediator of the promotility effects of PDA on the colon.

### 3.9 Safety Evaluation

To assess the safety profile of PCP during a 2-week treatment period, we monitored the body weight, major organ morphology, and serum parameters. As illustrated in Figure S8, none of the measured parameters showed marked changes across the groups. No notable histological abnormalities were found in any of the organs examined. These results indicated that administration of PCP at the tested doses for 2 weeks did not cause detectable toxicity in mice, suggesting its potential safety as a therapeutic.

## 4 Discussion

Accumulating studies have stressed the intimate correlation of constipation with the gut microbiota.^8,33^ Changes of gut microbiota in patients with chronic constipation are typified by a relative decline in probiotics (*e.g*., *Muribaculaceae* and *Bifidobacterium*) and a corresponding growth of potentially pathobionts (*e.g*., *Helicobacter*). These changes may affect colonic motility and secretory functions by modifying the abundance of physiologically active substances and the gut metabolic environment.^33^ Therefore, targeting the gut microbiota to restore a healthy microbial community represents a potential therapeutic strategy for constipation.

Herein, we found that the treatment with PCP significantly alleviated constipation in a mouse model, which might largely be attributed to its beneficial effects on intestinal functions. Mechanistically, microbiomics analysis revealed that PCP treatment reshaped the gut microbial community and enriched specific beneficial bacteria, particularly from *unclassified_f__Lachnospiraceae*. The family *Lachnospiraceae* is one of the most numerous and broadly present bacterial groups in the human colon tract.^34^ Members of this family can degrade various substrates, including cellobiose and fructose, and synthesize diverse metabolites, such as SCFAs,^35^ and they were shown to have anti-inflammatory, immunomodulatory, and homeostatic effects.^30,31^ *unclassified*_*f*__*Lachnospiraceae* abundance was positively associated with PDA levels. *In vitro* fermentation further supported the involvement of the gut microbiota in PDA production during PCP utilization. This result suggests that, under the influence of PCP, *unclassified_f__Lachnospiraceae* may serve as a key functional species associated with the production of this specific metabolite. Although PCP intervention significantly upregulated the production of specific branched-chain SCFAs (such as isobutyric and isovaleric acids), these metabolites exhibited weak or no significant correlations with constipation-related host phenotypes. This divergence suggests that the increase in these SCFAs likely reflects the general fermentation of PCP by the altered microbiota, representing metabolic byproducts rather than direct functional drivers of the colonic motility. In addition, compared with the constipated model group, the PDA level in lactulose-treated mice showed no significant change, whereas the levels of butyric acid, isohexanoic acid, and myristic acid were significantly elevated, indicating that PCP and lactulose exert their anticonstipation effects through distinct mechanisms.

Indeed, *Lachnospiraceae* strains have been reported to produce short (C2–C5) and long (C > 5) chain acids.^31^ PDA is an odd-chain saturated fatty acid,^30^ which can be produced by bacteria.^31,36^ Specifically, bacteria produce palmitic acid and stearic acid by means of the iterative condensation of malonyl-CoA with acetyl-CoA. Then, microbial populations convert palmitic acid or stearic acid to a hydroxyl fatty acid, after which decarboxylation occurs, yielding PDA or heptadecanoic acid, respectively.^26^ In addition to *Lachnospiraceae*, *Muribaculum* was also strongly correlated with PDA levels and reportedly harbors key PDA-biosynthetic genes (*fabf*, *fabk*, and *fabd*).^30^ Indeed, PDA production driven by polysaccharide utilization is widespread among gut commensals.^30^ For instance, a synbiotic combination of galactooligosaccharides and *Limosilactobacillus reuteri* could promote the synthesis of PDA from *Bacteroides acidifaciens*.^37^ Similarly, *Parabacteroides distasonis* utilizes inulin to yield PDA. Overall, the gut microbiota emerges as a principal contributor to intestinal PDA production.^38^ Therefore, the increase in PDA observed following PCP administration is likely to reflect coordinated metabolic activity of the gut microbial community rather than the activity of a single bacterial taxon. Future studies using bacterial isolates, strain-specific manipulation, or functional gene analysis will be necessary to determine the direct contribution of individual taxa, including Lachnospiraceae, to PCP-derived PDA production.

AQP3 and AQP4 are predominantly localized at the basolateral membrane of intestinal epithelial cells but differ in their distribution along the intestinal tract, with AQP4 showing a broader distribution than AQP3.^39^ In the present study, PCP significantly reduced the elevated expression of AQP4 in constipated mice, whereas AQP3 expression remained largely unchanged. This selective modulation may be partly attributed to their nonoverlapping distribution along the intestinal tract, as PCP’s action may preferentially target regions where AQP4 is more abundantly expressed, such as the proximal and midcolon, while AQP3 is less represented in these specific segments.^39–41^ Notably, PDA administration also reduced the elevated colonic AQP4 expression in constipated mice, suggesting that modulation of colonic water handling may accompany the prokinetic effect of PDA. However, the present study did not directly determine whether AQP4 is a downstream target of GPR40 signaling. Therefore, the observed changes in AQP4 should be considered an associated response to PCP/PDA treatment rather than evidence of a direct PDA–GPR40–AQP4 signaling cascade. Further studies using GPR40-specific genetic or pharmacological manipulation together with AQP4 assessment will be required to clarify this relationship.

It is widely recognized that GPR40 and GPR120, distributed in the endocrine cells of the colon, can be stimulated by free MLCFAs. However, GPR40, but not GPR120, is selectively involved in MLCFA-stimulated colonic motility. This is supported by the finding that enhanced muscle contraction triggered by MLCFAs was markedly inhibited by a GPR40 antagonist, whereas a GPR120 antagonist had no such effect.^15^ Consistent with the literature, we found that DC260126 (a GPR40 antagonist) completely blocked the effect of PDA on promoting colonic motility and alleviating constipation in mice. Meanwhile, we performed Ca^2+^ mobilization assays on Caco-2 cells. The results demonstrated that PDA treatment elicited a dose-dependent elevation of intracellular Ca^2+^, an effect that was markedly abolished by preincubation with DC260126. Collectively, these findings suggest that GPR40 is a critical mediator through which PDA exerts its promotility effects on the colon, although direct physical binding between PDA and GPR40 remains to be further confirmed by biophysical approaches.

Together, our results support a potential link between a microbiota-derived metabolite and a host receptor, in which PCP treatment enriches beneficial microbes associated with PDA production, thereby effectively accelerating intestinal transit and relieving constipation symptoms.

Although our findings support GPR40 as a key functional target of PDA, the precise molecular interaction between PDA and GPR40 warrants further structural validation. First, potential off-target effects of DC260126 as a small molecule antagonist cannot be excluded. Second, as molecular docking is merely a computational prediction, biophysical approaches such as surface plasmon resonance or isothermal titration calorimetry are required to directly confirm the binding between PDA and GPR40. Third, whether the *in vitro* findings fully recapitulate the physiological conditions within the complex intestinal microenvironment remains to be further validated by using gene-knockout animal models.

Regarding the safety of PCP, our 14-day toxicity study showed no significant histopathological alterations in major organs or serum biochemical abnormalities at the tested dosages. However, short-term observations cannot fully capture the potential risks associated with long-term administration. Prolonged PCP treatment may cause adaptive changes in the gut microbiota and alter microbial metabolite production, which could affect the sustained efficacy. In addition, persistent exposure to PDA and its interaction with GPR40 may potentially influence the receptor responsiveness and intestinal homeostasis. The long-term systemic exposure and safety of PCP-derived metabolites also remain unclear. Therefore, although the present study provides preliminary evidence supporting the short-term safety and efficacy of PCP, its long-term tolerability, sustained efficacy, and potential systemic effects should be further evaluated in well-designed chronic-dosing studies.

Collectively, this study demonstrates that PCP ameliorates loperamide-induced constipation in mice by stimulating intestinal motility and preserving the colonic water homeostasis. Our findings reveal that the therapeutic efficacy of PCP is deeply rooted in its capacity to modulate the gut microbiota, specifically by promoting the enrichment of *Lachnospiraceae*. This microbial shift facilitates the production of PDA, a key functional metabolite that is proposed to activate colonic GPR40 receptors to enhance gastrointestinal transit. By revealing a potential PCP–Lachnospiraceae–PDA–GPR40 regulatory axis, this research not only clarifies the molecular basis underlying the traditional use of *P. cyrtonema* but also supports further exploration for utilizing PCP as a precise prebiotic intervention or functional food ingredient for the management of chronic constipation.

## Supplementary Material



## Data Availability

Data will be made available on request.

## Notes

PCP characterization (Figure S1); AQP3 IF in mouse colon (Figure S2); PCP effects on defecation in female mice (Figure S3); gut microbiota analysis in the pharmacodynamic experiment (Figure S4); gut microbiota analysis following FMT (Figure S5); fecal metabolomics analysis (Figure S6); *in vitro* fermentation assay of PCP (Figure S7); and PCP safety evaluation (Figure S8) (PDF)

## Abbreviations

(PCP): *Polygonatum cyrtonema* polysaccharide
(FMT): Fecal microbiota transplantation
(PDA): Pentadecanoic acid
(GPR40): G-protein coupled receptor 40
(SEM): Scanning electron microscope
(PBS): Phosphate-buffered saline
(IF): Immunofluorescence
(AQP): Aquaporin
(SD): Standard deviation
(HPSEC): High-performance size-exclusion chromatography
(ASVs): Amplicon sequence variants
(PCoA): Principal coordinate analysis
(LDA): Linear discriminant analysis
(LEfSe): Linear discriminant analysis effect size
(NMDS): Nonmetric multidimensional scaling
(PCA): Principal component analysis
(PLS-DA): Partial least-squares-discriminant analysis
(SCFAs): Short-chain fatty acids
(MLCFAs): Medium- to long-chain fatty acids
(AST): Aspartate aminotransferase
(ALT): Alanine aminotransferase
