# Supplementary material for: Polygonatum cyrtonema Polysaccharide Ameliorates Functional Constipation in Mice via the Gut Microbiota–Pentadecanoic Acid–GPR40 Axis
Source: J Agric Food Chem. 2026 Oct 20;74(43):33918–33. doi: 10.1021/acs.jafc.6c06937 (PMC13741903; doi:10.1021/acs.jafc.6c06937)
Supplement: Supplementary file 1 [file jf6c06937_si_001.pdf]

Supporting Information

Polygonatum cyrtonema polysaccharide ameliorates functional constipation in mice via the gut microbiota-pentadecanoic acid-GPR40 axis

Xiaoying Liu[a], Zhongyu Zhang[a], Yuanyuan Fang[a], Beixin Yuan[a], Huilong Lian[a], Wenxin Yin[a], Jinming Zhang[b], Qin Yuan[b,*], Shengpeng Wang[a,c,*]

[a]*State Key Laboratory of Mechanism and Quality of Chinese Medicine, University of Macau, Macao SAR 999078, China*

[b]*School of Pharmacy, Chengdu University of Traditional Chinese Medicine, Chengdu 611137, China.*

[c]*Macao Centre for Research and Development in Chinese Medicine, Institute of Chinese Medical Sciences, University of Macau, Macao SAR 999078, China*

[*]*Corresponding authors*

*Corresponding to: swang@um.edu.mo (S. Wang) and yuanqin@cdutcm.edu.cn (Q. Yuan).

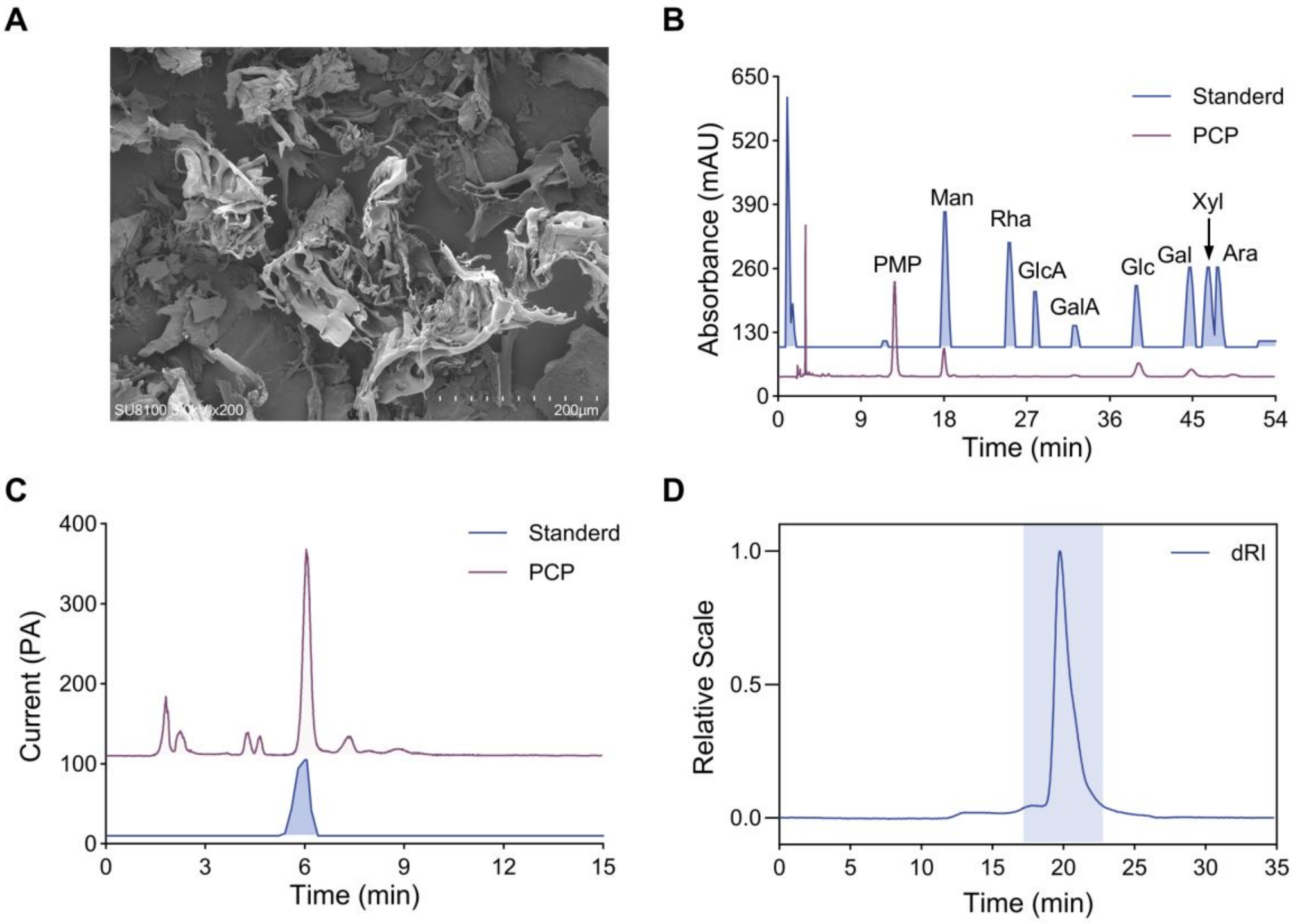

Fig. S1. **Characterization of PCP.** (A) Representative scanning electron microscopy (SEM) images showing the surface morphology of PCP (200× magnification). (B) HPSEC chromatograms of PCP. (C-D) Monosaccharide composition chromatogram.

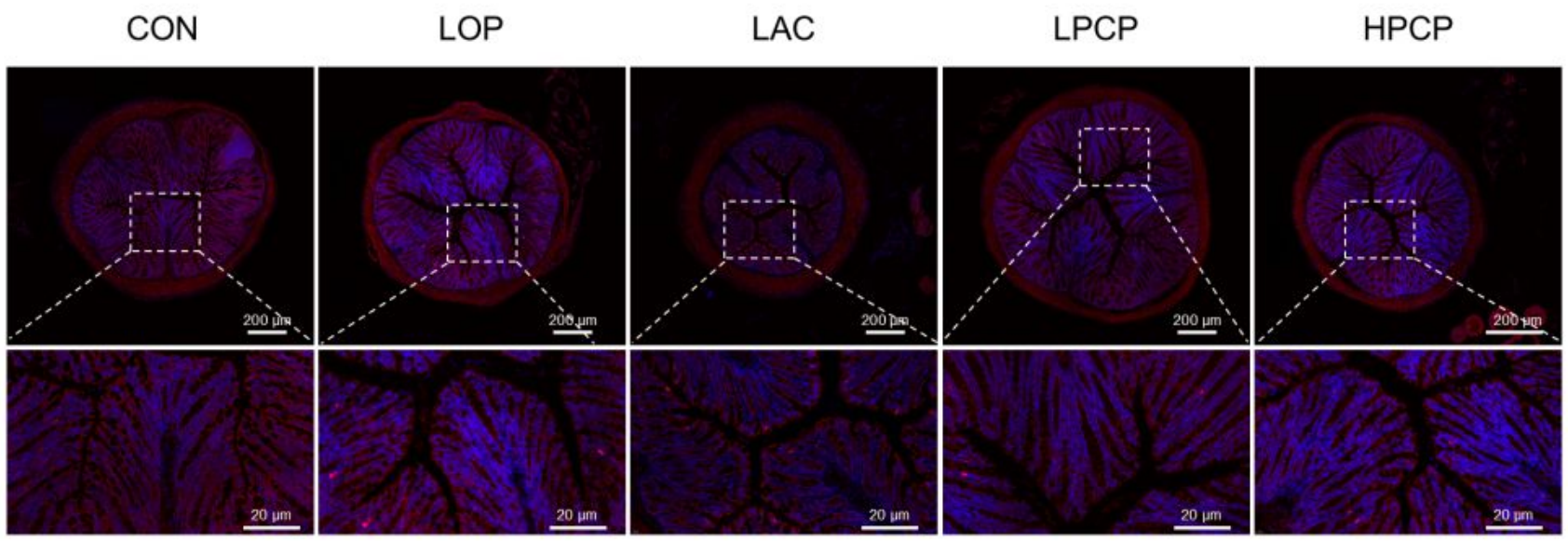

Fig. S2. Representative IF images showing the effect of PCP treatment on colonic AQP3 expression in mice (*n* = 3). Scale bars: 200 μm (upper), 20 μm (lower).

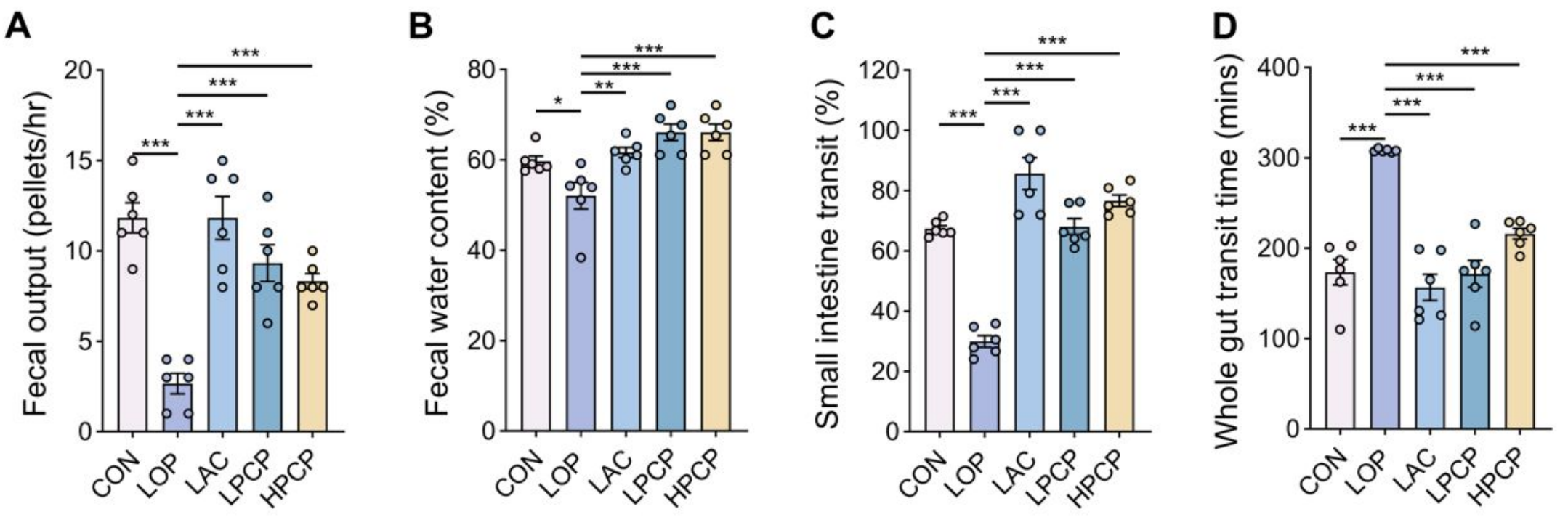

Fig. S3. PCP increased fecal output, fecal water content and promoted gastrointestinal motility in female constipated mice. (A) Fecal output. (B) Fecal water content. (C) Small intestine transit rate. (D) Whole gut transit time. Data represent the mean ± SD (*n* = 6). **P* < 0.05, ***P* < 0.01, ****P* < 0.001.-

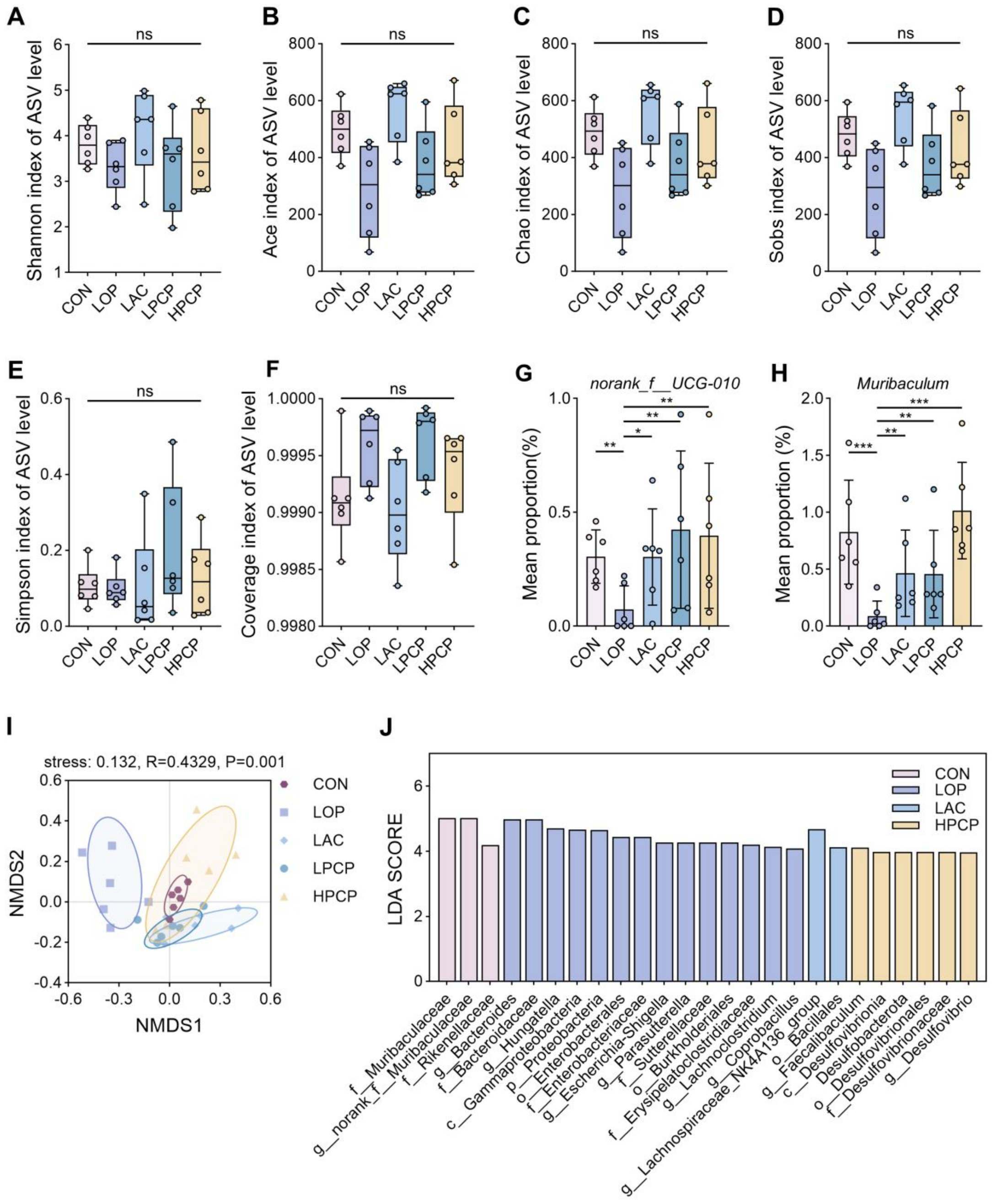

Fig. S4. PCP ameliorated gut microbiota dysbiosis. (A-F) α-diversity represented by the Shannon (A), Ace (B), Chao (C), Sobs (D), Simpson (E), and Coverage (F) indices with one-way ANOVA test. (G-H) Relative abundance of *norank_f__UCG 010* (G) and *Muribaculum* (H). (I) NMDS analysis at the ASV level. (J) Analysis of differences in the microbial taxa among groups was performed using the linear discriminant analysis (LDA) effect size (LEfSe) (LDA score ≥ 4.0). Data represent the mean ± SD (n = 6). *$P < 0.05$, **$P < 0.01$, ***$P < 0.001$.

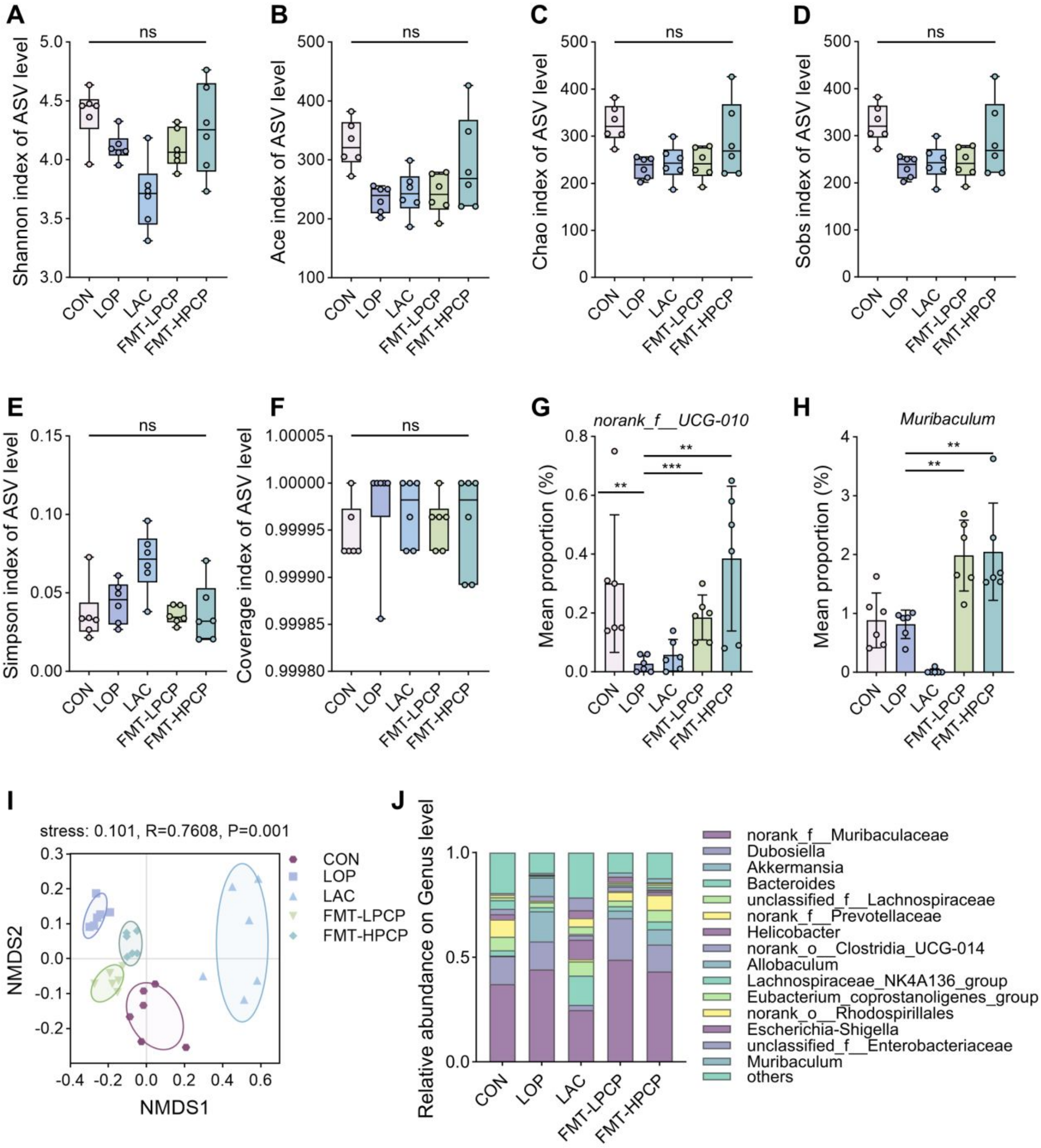

Fig. S5. FMT treatment ameliorated gut microbiota dysbiosis. (A-F) α-diversity represented by the Shannon (A), Ace (B), Chao (C), Sobs (D), Simpson (E), and Coverage (F) indices with one-way ANOVA test. (G-H) Relative abundance of *norank_f__UCG 010* (G) and *Muribaculum* (H). (I) NMDS analysis at the ASV level. (J) Relative abundance of gut microbiota at the genus level. Data represent the mean ± SD (n = 6). $^*P < 0.05$, $^{**}P < 0.01$, $^{***}P < 0.001$.

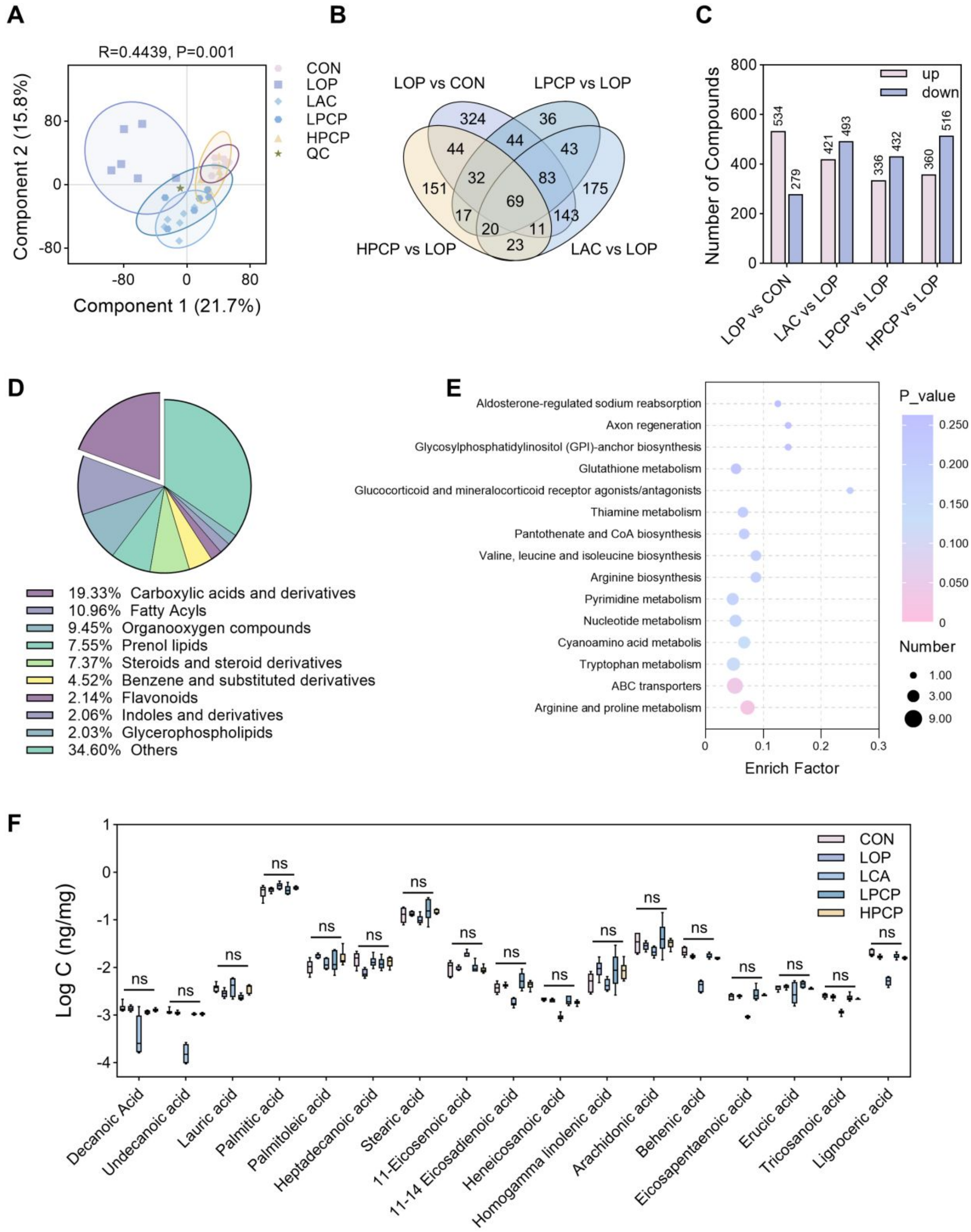

Fig. S6. Effect of PCP on fecal metabolomics in constipation mice. (A) PLS-DA score plot of metabolomic features among groups. (B) Petal Venn diagram of metabolic sets among different groups. (C) The number of differentially abundant metabolites among groups. (D) HMDB compound classification of differential metabolites. (E) KEGG pathway enrichment of differentially abundant metabolites. The circles represent different KEGG pathways. The size of the circle represents the number of differentially abundant metabolites in this pathway, and the color represents the *p* value,

which is the significance level in enrichment analysis. (F) Quantification of MLCFAs in mouse feces. Data represent the mean ± SD ($n = 6$). *$P < 0.05$, **$P < 0.01$, ***$P < 0.001$; ns, not significant.

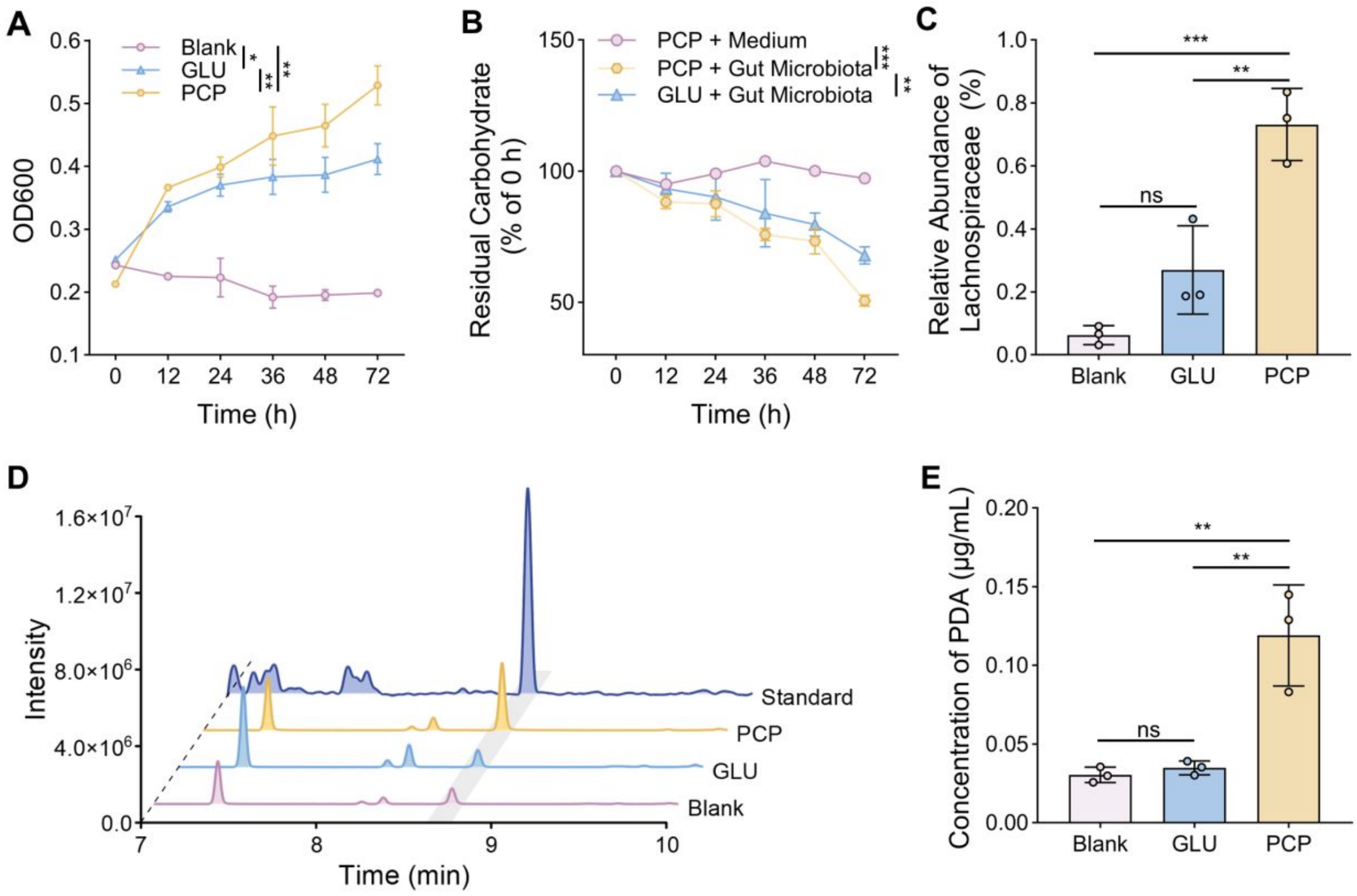

Fig. S7. Effects of PCP on gut microbiota growth, carbohydrate consumption, and PDA production during *in vitro* fermentation. (A) Growth curves of gut microbiota in Blank, GLU, and PCP groups evaluated by OD_{600} over 72 h. (B) Residual carbohydrate concentration (% of 0 h) during 72 h of *in vitro* fermentation. (C) Relative abundance of *Lachnospiraceae* after 72 h of fermentation in Blank, GLU, and PCP groups. (D) Representative GC-MS extracted ion chromatograms (EIC) of PDA in standard and fermentation broth samples at 72 h. (E) Quantification of PDA concentration (μg/mL) after 72 h of fermentation. Data represent the mean ± SD ($n = 3$). *$P < 0.05$, **$P < 0.01$, ***$P < 0.001$; ns, not significant.

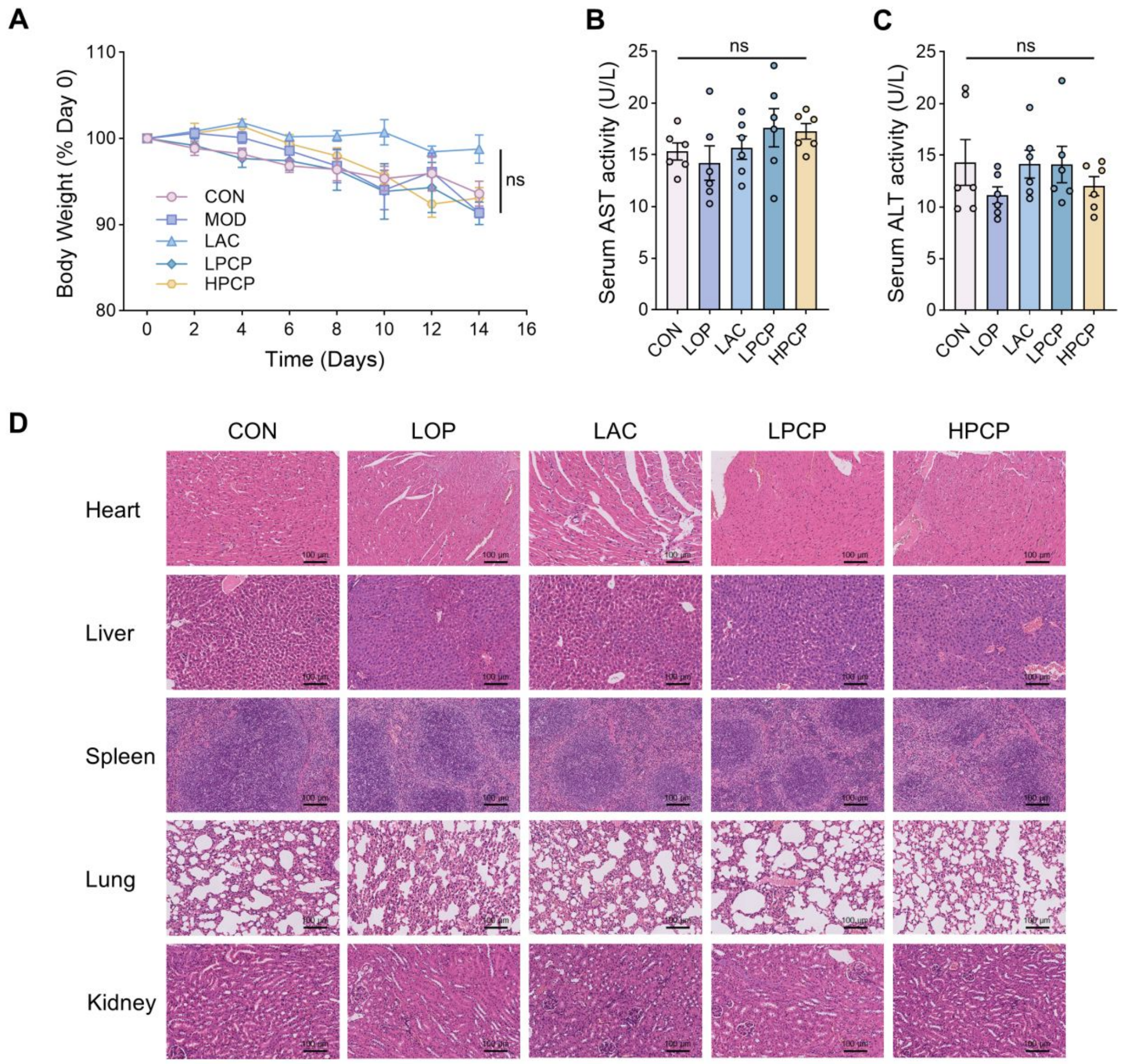

Fig. S8. Safety evaluation of PCP. (A) Body weight of mice treated with PCP. (B) Serum aspartate aminotransferase (AST) activity. (C) Serum alanine aminotransferase (ALT) activity. (D) Representative H&E staining of heart, liver, spleen, lung, and kidney tissues from the five groups. Scale bars: 100 µm. Data represent the mean ± SD (*n* = 6 for A-C, and *n* = 3 for D). ns: not significant.
